# The Peripheral Flicker Illusion

**DOI:** 10.1177/2041669517747891

**Published:** 2017-12-20

**Authors:** Hiroyuki Ito, Tomomi Koizumi

**Affiliations:** Faculty of Design, Kyushu University, Fukuoka-shi, Japan; Research Center for Applied Perceptual Science, Kyushu University, Fukuoka-shi, Japan; Graduate School of Design, Kyushu University, Fukuoka-shi, Japan

**Keywords:** illusion, color, peripheral vision, rod-cone interaction

## Abstract

A new illusion is reported. A visual object suddenly appearing on a red background sometimes causes an impression of flicker or double flash. In Experiment 1, a red, green, or blue object was presented on a red, green, blue, or gray background. Participants evaluated the illusion strength in reference to the physical flicker of a gray object presented in central vision. The results show that the green or blue object presented on the red background caused the illusion. In Experiment 2, the effect of retinal eccentricity on the illusion was tested. The results showed that the illusion was weak in central vision but became stronger as the retinal eccentricity of the objects’ presentation increased. In Experiment 3, optimal luminance conditions for the illusion were explored with the green and blue objects. The illusion was strong when object luminance was lower than background luminance and the optimal luminance for the blue object was lower than that for the green object. We propose a tentative theory for the illusion and discuss its cause.

## Introduction

Visual illusions producing a double flash impression from one physical visual flash have previously been reported. For example, the visual illusion of double (or triple) flash is induced by multiple auditory beeps (Shams, Kamitani, & Shimojo, 2000), two brief tactile stimuli (Violentyev, Shimojo, & Shams, 2005), or two successive visual flashes presented with the target flash (Chatterjee, Wu, & Sheth, 2011; Wilson, 1987; Wilson & Singer, 1981). These illusions commonly include two components: a briefly presented visual target and a simultaneously presented inducer that *flashes* multiple times. The single reported exception is the double-flash illusion described by Bowen, Markell, and Schoon (1980) and Bowen, Mallow, and Harder (1987). Their stimulus included a briefly presented visual target but not a flickering inducer. They demonstrated that a briefly presented light pulse is seen as two flashes when presented 0.1 to 0.3 seconds after a light field vanishes. In this article, we present a new illusion of double flashes or flicker. The illusion is much simpler than those described previously in that the stimulus does not include a brief presentation of the target—that is, the target does not need to disappear shortly after its appearance—and there is no flickering inducer. Therefore, the illusion can be induced even by slideshow software (for a demonstration, see online PowerPoint file). We call this phenomenon *the peripheral flicker illusion.*

**Figure 1.**
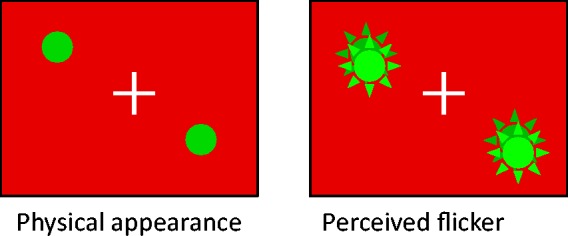
Schematic illustration of the phenomenon. As a typical example, when green objects suddenly appear on a red background, the objects are seen to flicker or flash twice. There is no need for a flickering inducer or brief presentation of the target.

Figure 1 shows a schematic illustration of the illusion. We have observed that when colored objects suddenly appear on a red background, they are sometimes seen to flicker or flash twice, occasionally followed by a small amplitude of trembles in brightness. Although the perceptual flicker is very robust in peripheral vision, if observers fixate the location where the object is to appear (i.e., see the object in central vision), they may notice that the suddenly-appearing object actually never flickers (see Figure 1). Surprisingly, despite its simplicity, this effect has not previously been described by researchers, to the best of our knowledge. In three experiments, this study sought to describe the present phenomenon in detail and to determine the favored conditions for its occurrence.

To document the illusory effect, we employed a method of magnitude estimation with a reference object that physically flickered at the moment of appearance. In Experiment 1, the effect of color combination was investigated to find the necessary color conditions for this illusion. In Experiment 2, the effect of retinal eccentricity of object presentation was tested. In Experiment 3, optimal luminance conditions for the illusion were explored. The results are discussed in the context of a tentative theory.

## Experiment 1

Experiment 1 explored necessary conditions for the illusion with regard to color combinations between objects and a background, keeping the luminance relationship constant.

### Method

#### Participants

Nine naïve volunteers and the second author (ranging from 21 to 34 years in age) participated in Experiment 1. They all had normal or corrected-to-normal visual acuity. Before the experiment, informed consent was obtained. This study was approved by the local ethics committee of Kyushu University.

#### Apparatus and stimuli

All the stimuli were produced by a computer (Dell, Inspiron 400) and displayed on an organic light-emitting diode (OLED) display (Sony, PVM-2541). Although one video frame of the OLED display was 16.7 ms (refreshing at 60 Hz), the physical duration of image exhibition was about 7.5 ms within one frame (see detailed description of the display characteristics in Ito, Ogawa, & Sunaga, 2013). CIE 1931 *xy* chromaticity values of the RGB primaries and gray were red (0.6766, 0.3230), green (0.1877, 0.7239), blue (0.1417, 0.0500), and gray (0.3117, 0.3229) measured with a spectral radiometer (Konica Minolta, CS-2000). The display was treated as a 1920 (horizontal) × 1080 (vertical) pixel matrix.

**Figure 2.**
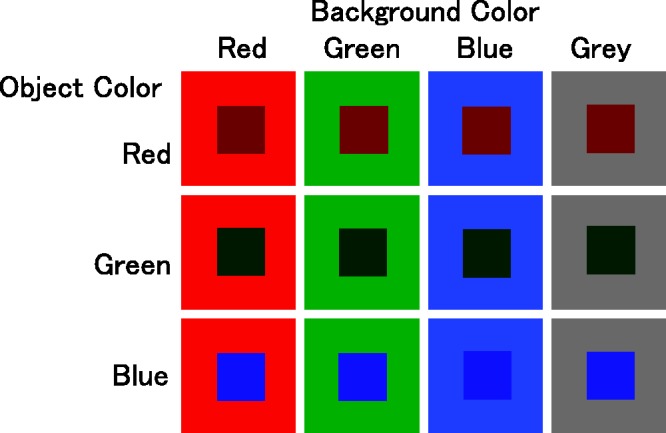
Color combinations tested in Experiment 1. Luminance was virtually the same for all background colors (6.0 cd/m^2^) and for all object colors (2.0 cd/m^2^).

**Figure 3.**
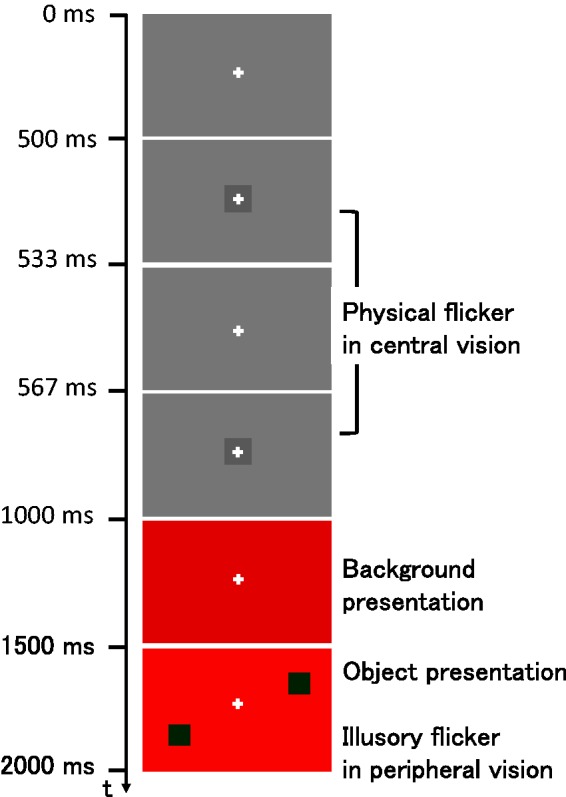
Schematic illustration of the time course of a trial sequence. A 60-frame duration equals 1,000 ms. First, a gray square was presented on a gray background with a physical flicker. Next, a colored background was presented, followed by colored squares in the lower left and upper right positions or the upper left and lower right positions. These squares did not physically flicker but were sometimes seen as flickering at the time of their presentation.

Tested background colors were red, green, blue, and gray. The luminance of all background colors was virtually the same (red: 6.0, green: 5.9, blue: 6.0, and gray: 6.1 cd/m^2^). Tested object colors were red, green, and blue. The luminance of all object colors was the same at 2.0 cd/m^2^. Thus, there were 12 object-background color combinations with the luminance relationship kept constant (see Figure 2). In addition, as a reference, we presented a gray object (2.0 cd/m^2^) at the beginning of each trial. Both the colored objects and the gray reference object were squares of 100 pixels per side. The squares subtended 2.7° of visual angle when the object was presented in the center of the screen, where a fixation cross was placed. In Experiment 1, the tested colored objects were presented in lower left and upper right positions from the fixation point (26.6° orientation from the horizontal, see Figure 3), or in upper left and lower right positions, avoiding blind spots. The center of each object was 11.9° from the center of the fixation cross; that is, retinal eccentricity was 11.9°, although the reference gray object was always presented in the center.

#### Procedure

Participants viewed the display at a distance of 60 cm. Their heads were restricted by a chinrest. Within a session, the experimenter first explained the task and showed the stimulus displays to the participant.

Figure 3 shows the time course of one-trial sequence. First, a gray background was presented with a white fixation cross in the center of the screen. The fixation cross remained visible throughout the trial. After 500 ms, a dark gray object (2.0 cd/m^2^) appeared in the center and was displayed for two frames (33.3 ms), then disappeared. Two frames (33.3 ms) after that, the dark gray object appeared again. This sequence produced physical flicker of the gray object. From a preliminary experiment, we knew that a gray object appearing in central vision on a gray background did not cause illusory flicker. After presentation of the gray object for 433 ms, a colored background was presented for 500 ms followed by the colored object for 500 ms. Participants evaluated the flicker impression of the colored objects relative to the physical flicker of the gray object. Participants assigned a value of 10 if the flicker appeared to be the same strength as that of the reference stimulus, a value of 0 if no flicker was perceived, and intermediate values between 1 and 9 or above 10 to represent perceived flicker that was weaker or stronger than the reference, respectively. Participants input their selected values using a computer keyboard.

A single session included all 12 color combinations, each presented once. After one session of training, six experimental sessions were conducted. Stimulus order was randomized within a session.

### Results and Discussion

**Figure 4.**
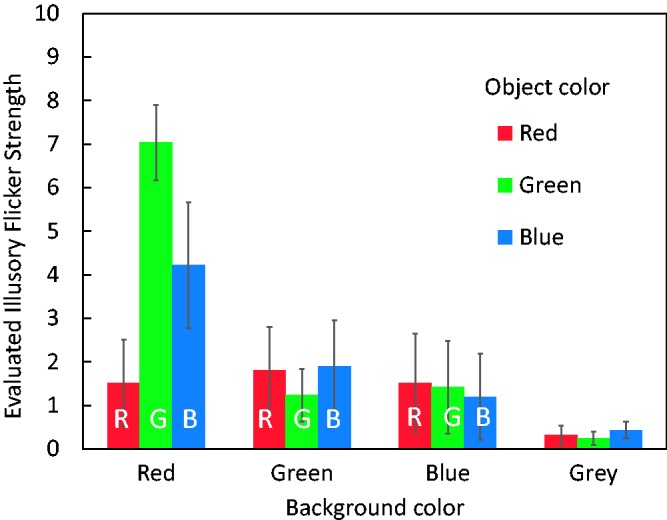
Results of Experiment 1. The vertical axis indicates evaluated values of illusion strength averaged over the 10 participants’ responses. A value of 10 indicates the same strength in flicker impression as that of the physical flicker of the gray object on a gray background viewed in central vision. Error bars indicate standard errors.

Figure 4 shows the averaged values of illusion strength evaluated under the 12 color-combination conditions. The illusion was the strongest when a green object appeared on a red background. As the referenced physical flicker occurred between the object and background with a large luminance ratio (6 cd/m^2^ to 2 cd/m^2^) producing sufficient flicker impressions, it is thought that the illusory flicker with the average evaluated value of 7.033 was also clearly perceived. The next-strongest color combination was blue object–red background.

Statistical analyses were performed in OriginPro software (OriginLab Corp.). A repeated-measures two-way analysis of variance (ANOVA) was conducted to test the effects of object and background colors. When Mauchly’s test rejected the sphericity of the data, the degrees of freedom were adjusted by the Huynh–Feldt epsilon. There were significant main effects of object color, *F*(2, 18) = 7.516, *p* = .0042, $\eta_{p}^{2}$^ ^= 0.4550, and background color, *F*(2.07, 18.65) = 9.000, *p* = .0017, $\eta_{p}^{2}$^ ^= 0.4999. The interaction was also significant, *F*(2.75, 24.74) = 16.684, *p* < .0001, $\eta_{p}^{2}$^ ^= 0.6495. Multiple comparisons (Scheffe’s method, α level = .05) to test the object color effect revealed that, under the red background condition, there were significant differences between the green–red, green–blue, and red–blue object color pairs. No significant difference between an object color pair was found under the green, blue, and gray background conditions. Multiple comparisons to test the background color effect also showed that under the green object-color condition, there were significant differences between the red–blue, red–green, and red–gray background color pairs. Under the blue object-color condition, there were significant differences between the red–gray and red–blue background color pairs.

These statistics could be interpreted as follows: (a) the red background produced a strong effect only for the green or blue object but not for the red object and (b) the blue, green, and gray backgrounds did not produce large flicker impressions. In summary, the illusion mainly arises when a green or blue object appears on a red background.

## Experiment 2

Experiment 2 investigated the effect of retinal eccentricity of object presentation. As both green and blue objects caused the illusion in Experiment 1, rods may play a contributing role. If so, the illusion would be stronger for objects viewed peripherally and would not occur for objects viewed directly.

### Methods

**Figure 5.**
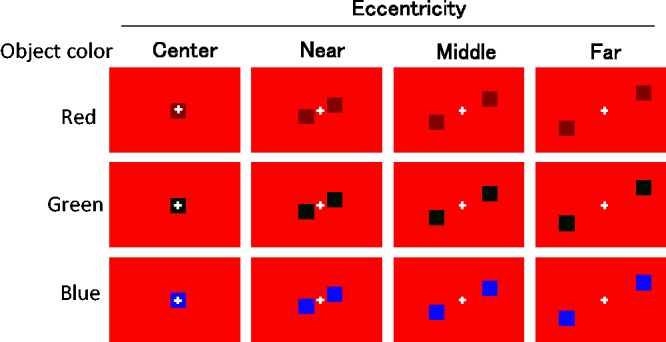
Schematic illustration of the conditions. In the center condition, only one colored object appeared.

As shown in Figure 5, the stimulus conditions were combinations of object colors and retinal eccentricities. Within a trial, object color was red, green, or blue (2.0 cd/m^2^). The background color was always red (6.0 cd/m^2^). Eccentricity was defined as the retinal distance between the center of the fixation cross and center of the object. Retinal eccentricities were 0°, 6.0°, 11.9°, or 17.6°, we refer to these as the *center*, *near*, *middle*, and *far* conditions, respectively. All other methods, and the same participants, were used as in Experiment 1.

### Results and Discussion

**Figure 6.**
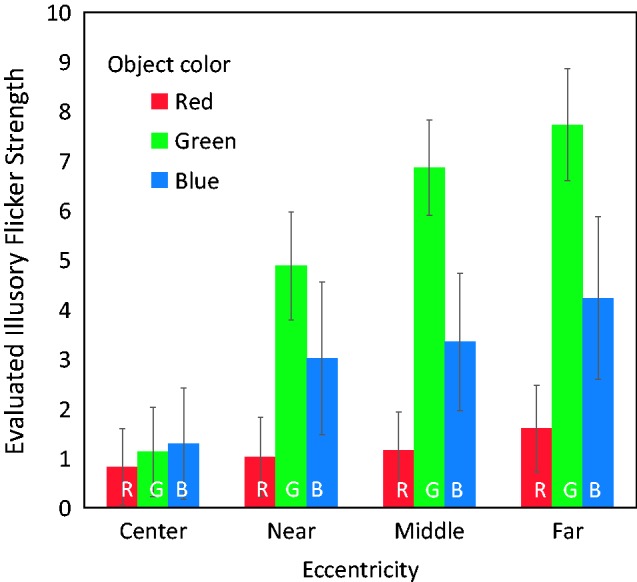
Results of Experiment 2. The vertical axis indicates evaluated values of the illusion strength, averaged over all 10 participants’ responses. Evaluated values of illusory-flicker strength were larger in peripheral vision for the green and blue objects and were small across the tested eccentricities for the red object. Error bars indicate standard errors.

As shown in Figure 6, a strong illusion occurred only for the green and blue objects. The red objects produced little effect, as was seen in Experiment 1. Also in the center condition, the illusion was weak regardless of object color. The effect of the fixation cross itself was not important in the center condition. We unofficially observed that the illusory flicker seldom arose in central vision, also when the cross was absent. In the green and blue object conditions, the illusion tended to become stronger as eccentricity increased. The average rating estimated for the green object was 7.733 in the far conditions.

A repeated-measures two-way ANOVA was conducted to test the effects of object color and eccentricity. Mauchly’s test again rejected the sphericity of the data, so the degrees of freedom were adjusted by the Huynh–Feldt epsilon. There were significant main effects of object color, *F*(2, 18) = 15.237, *p* = .0001, $\eta_{p}^{2}$^ ^= 0.6287, and eccentricity, *F*(1.76, 15.80) = 15.564, *p* = .0003, $\eta_{p}^{2}$^ ^= 0.6336. The interaction was also significant, *F*(3.69, 33.22) = 13.599, *p* < .0001, $\eta_{p}^{2}$^ ^= 0.6018. Multiple comparisons (Scheffe’s method, α level = 0.05) to test the eccentricity effect revealed that, for the green object, there were significant differences between the center-near, center-middle, center-far, and near-far eccentricity pairs. For the blue object, there was a significant difference between the center-far eccentricity pair. For the red object, no significant eccentricity effect was found. Multiple comparisons to test the object-color effect revealed that, for the far eccentricity condition, there were significant differences between the green–blue, green–red, and blue–red object color pairs. For the middle eccentricity condition, there were significant differences between the green–blue and green–red object color pairs. For the near eccentricity condition, there was a significant difference between the green–red object color pair. For the center condition, there was no significant difference between the object color pairs. These statistics confirm that this illusion mainly arises when an observer views a green or blue object on a red background in peripheral vision.

It is possible that misperception of an object onset in peripheral vision produces the flicker illusion. However, because little illusory flicker occurred for red objects, as opposed to green objects, even in the far peripheral visual field, the illusion cannot be triggered solely by a sudden luminance change. As both green and blue objects appearing in peripheral vision produced a strong illusion, rods are again implicated as potential contributors to the effect. In Experiments 1 and 2, the blue object produced a weaker illusion than did the green object. However, as will be shown in Experiment 3, the luminance conditions in Experiments 1 and 2 were more favorable for producing the illusion with the green object.

## Experiment 3

Experiment 3 investigated favored object-luminance conditions for this illusion. Whereas Experiments 1 and 2 tested the illusion with fixed object luminance, in Experiment 3 we manipulated object luminance. In some conditions, object luminance was higher than background luminance.

### Methods

**Figure 7.**
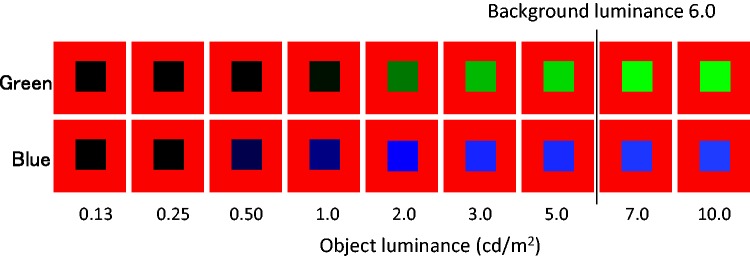
Tested conditions in Experiment 3. Object luminances of 7.0 and 10.0 cd/m^2^ exceeded the luminance of the red background.

There were two object colors (green and blue) and nine object luminances (0.13, 0.25, 0.5, 1.0, 2.0, 3.0, 5.0, 7.0, or 10.0 cd/m^2^), as shown in Figure 7. Retinal eccentricity of object was constant at 11.9°, as in Experiment 1. The background color was always red (6.0 cd/m^2^). The 18 conditions were presented in random order within one experimental session. Each participant completed six sessions. Participants were nine naïve observers and the second author. Only two participants had not taken part in Experiments 1 and 2. All other methods were the same as in Experiments 1 and 2.

### Results

**Figure 8.**
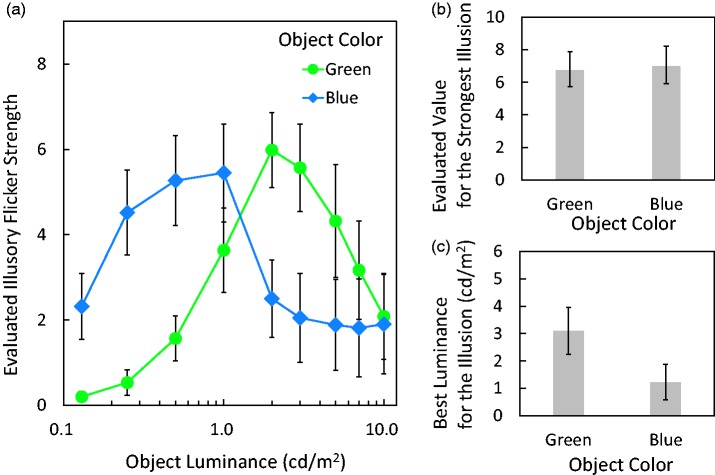
The results of Experiment 3. (a) Evaluated illusion strength as a function of object luminance. Green symbols show the illusion strength for green objects, averaged over all 10 participants. Blue symbols show the data for blue objects. (b) Evaluated values for the strongest illusion. The highest evaluation values for each participant under each object-color condition were averaged. (c) Optimal luminance for the illusion. The luminances at which the highest evaluation value was acquired were averaged. Error bars indicate standard errors.

As shown in Figure 8(a), the distribution of evaluated illusion strength averaged over all 10 participants formed an inverted U shape, that is, low at the lowest and highest luminance conditions, with a peak in between. The object luminance that produced the strongest effect for the green object was higher than that for the blue object.

A Friedman test was conducted for each object-color condition. There was a significant effect of luminance for the green object (χ^2 ^= 50.1333, *df* = 8, *p* < .0001) and for the blue object (χ^2 ^= 39.34, *df* = 8, *p* < .0001). Illusion strength changed, depending on the object’s luminance, under each object-color conditions. A difference in illusion strength between the green and blue objects was tested by comparing the peak values of ratings. The highest values in each participant’s responses for the green object were a mean of 6.8 (*SE* = 1.0752) and a median of 6.3333. The peak values for the blue object were a mean of 7.0667 (*SE* = 1.136) and a median of 5.5833 (see Figure 8(b)). The difference was not significant, paired *t* test: *t*(9) = − 0.8817, *p* = .4009, two-tailed, *r* = .28; Wilcoxon signed-rank test: *Z* = −0.7114, *p* = .4766, *r* = .1591). In Experiments 1 and 2, the blue object seemed to produce a weaker illusion than did the green object. However, under certain luminance conditions, the illusion strength elicited by the blue object was as strong as that of the green object. A difference in optimal luminances of the green and blue objects was tested by comparing the luminances where the peak evaluated values were acquired for each participant. The luminance that produced the strongest effect for the green object had a mean of 3.1 cd/m^2^ (*SE* = 0.8622) and a median of 2.5 cd/m^2^. The optimal values for the blue object were a mean of 1.225 cd/m^2^ (*SE* = 0.6492) and a median of 0.5 cd/m^2^ (see Figure 7(c)). The difference was significant, paired *t* test: *t*(9) = 4.5503, *p* = .0014, two-tailed, *r* = .84; Wilcoxon signed-rank test: *Z* = 2.7557, *p* = .0020, *r* = .6162.

## General Discussion

In three experiments, a new phenomenon called the peripheral flicker illusion was reported. Experiment 1 showed that green or blue objects appearing on a red background were seen to flash twice or to flicker. Experiment 2 showed that the illusion occurred only in peripheral vision. Experiment 3 revealed that the illusion arose when the luminance of the green or blue object was lower than that of the red background, and that the best luminance for the illusion was lower for the blue object than for the green object.

One may argue that the flicker illusion is an artifact caused by the OLED display, which physically displayed the image for 7.5 ms in one video frame (16.7 ms). Thus, physical flicker always existed during the image presentation. However, the illusion also arises with a liquid crystal display (LCD) that produces little flicker. Therefore, the illusion arises from the characteristics of the human visual system, not of the display. We used an OLED display for its advantage of less fluctuation of luminance and color as a function of screen position or viewing direction for its purity of the primary colors (especially at lower luminances) and for the short rise and fall time in luminance change. In this study, stimuli with luminance less than 1.0 cd/m^2^ were used, making LCD a relatively poor choice for color purity.

**Figure 9.**
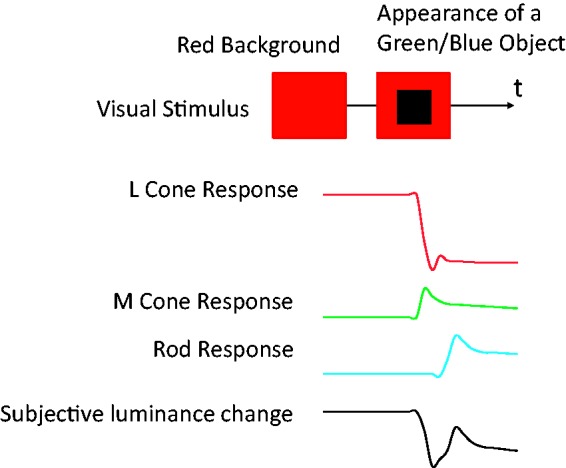
A schematic illustration of the rod-cone latency difference model. The depicted responses are simulations for rods and cones with receptive fields within the green or blue object. When a green or blue object appears, the L-cone responses decrease while M or S cone responses increase. The transient component of the L-cone responses constitutes a trough in subjective luminance change. The transient component of the delayed rod responses constitutes a subsequent peak. The red background (or L-cone activities) may enhance the dissociation of the two transient signals in timing. These peak and trough in subjective luminance change may constitute a flicker impression. Object color detection by S cones preceding the delayed rod response might affect the illusory flicker impression.

We think that the rod response to an appearing object is one of the important factors producing the illusion. There are two reasons for this view. First, a red object did not cause the effect, while a green or blue object did (Experiments 1 and 2). Second, the effect was present with peripheral vision but not central vision (Experiment 2). At present, we can only present a tentative theory for the illusion incorporating the rod responses (see Figure 9 as a qualitative model). An appearing green or blue object causes cone and rod onset responses while, at the same time, the vanishing red background causes L-cone offset responses. However, these changes in rod and cone responses do not occur simultaneously: The rod response is thought to lag the cone response by dozens of milliseconds (e.g., 29 ms, Cao, Lee, & Sun, 2010; 52 ms, Anstis & Macleod, 2015). We hypothesize that the transient signals of cone and delayed rod responses produce the flickering impression.

We conducted unofficial observations using a green object appearing on a black background. In this case, both cone and rod responses rise when the object appears. According to the aforementioned theory, it is possible that the cone and delayed rod responses would produce some flicker effect; however, the flickering impression was not apparent. Because the time lag between cone and rod responses is usually not perceived, even when an object suddenly appears in mesopic and peripheral vision, the onset signals from rods and cones should be integrated somehow. The present illusion may be an example where the integration is broken. Contrarily, when a green or blue object appeared on a red background that was continuously presented, causing the object to look orange or magenta in color by additive color mixture (the object was perceived as somewhat transparent), surprisingly, this condition produced a perceptual flicker. In this condition, physically, green or blue light was simply added to red light. The rise of rod and cone activities from the black background does not clearly produce the illusion, while that from the red background does. This might indicate that L-cone activities interact with rod activities (Shapiro, 2002) and break the integration of rod and cone onset signals, compounding the delay of the rod response.

In Experiment 3, we acquired different optimal luminances for the green and blue objects to produce the illusion. The luminance described here is photopic and was calculated with the human photopic luminous efficiency function, having peak sensitivity at 555 nm and lower sensitivity for blue light, ignoring rod activities. Thus, when blue and green are set at the same photometric luminance, the blue is much brighter for rods that have peak sensitivity at 496 nm and respond to blue and green light. This could be one reason for the difference in most effective luminance for the effect between blue and green lights. We calculated the luminances, employing the CIE 1964 10° color matching functions (Wyszecki & Stiles, 1982). The best luminances, that is, 3.1 cd/m^2^ in green and 1.2 cd/m^2^ in blue, correspond to 3.34 cd/m^2^ and 2.28 cd/m^2^ in the 10° color matching functions, respectively. We also calculated scotopic luminances for the best luminances (Wyszecki & Stiles, 1982). Photopic luminance of 3.1 cd/m^2^ in green or 1.2 cd/m^2^ in blue displayed on our monitor corresponds to 8.43 scotopic cd/m^2^ or 20.97 scotopic cd/m^2^, respectively. Thus, as for rods, the best blue luminance is much “brighter” than the best green luminance. If the rod responses solely determine the illusion strength, (a) the best conditions for the blue and green objects should elicit roughly equal rod responses because the illusion strength was not different under the two conditions and (b) the illusion strength should reflect scotopic luminances rather than photopic luminances. However, the scotopic luminances under the best conditions for the green and blue objects were not similar as noted earlier and were not the best parameter predicting the illusion strength (luminances calculated for a 10° field were better). Thus, it is not likely that the rod responses solely determine the illusion strength.

It is also possible to argue that S cones, not rods, contribute to the illusion. In some circumstances, S cones have been shown to contribute to luminance (Lee & Stromeyer, 1989; Stockman, Macleod, & DePriest, 1991), which could induce a flicker impression. According to Stockman et al. (1991), there are two S-cone processes with different temporal properties, that is, low- and high-frequency channels corresponding to chromatic and achromatic (luminance) pathways, respectively (although responses from S cones are negatively connected to the luminance pathway). In addition, S-cone signals cause a delay in perceptual responses depending on the state of adaptation, for example, by 23 to 44 ms (Anderson, Husain, & Somner, 2008; Bompas & Sumner, 2008; Ripamonti, Woo, Crowther, & Stockman, 2009), which is comparable to the delay of rod responses. Therefore, it is possible that S-cone signals substitute for rod signals in our theory.

However, we think that rods are more important for the effect than S cones for the following reasons. First, S cones have a peak at 419 nm in light absorbance and show little absorbance to over-500 nm light measured with microspectrophotometry (Dartnall, Bowmaker, & Mollon, 1983). Our OLED display emits mainly over 500 nm light as green light (see Figure 10 in Ito et al., 2013). Therefore, it is difficult to think that S cones induce the present effect similarly for the green light and the blue light. Rods are considered to have peak sensitivity around 496 nm (Dartnall et al., 1983) and would respond well to both blue and green lights in our display. Second, distribution of S cones on the retina is different from that of rods. While rods have their peak density at around 20° retinal eccentricity (Osterberg, 1935), S cones have the peak at around 1° (see Calkins, 2001 for a review). The S-cone density rapidly decreases as retinal eccentricity increases beyond 1°. Thus, the results of Experiment 2 (the effect increased as the stimulated retinal eccentricity increased from 0° to 17.6°) fit the rod versus cone hypothesis.

Currently, we do not have decisive evidence acquired from experiments to exclude the possibility of the contribution of S cones to the present effect. Independent control of the rod or S-cone activities may be needed to solve the problem. This should be tackled in the future. Our next series of experiments will investigate the predictions and the aforementioned observed phenomena to thoroughly modify the tentative theory.

## Supplementary Material

Supplementary material
