# Supplementary material for: The Peripheral Flicker Illusion
Source: Iperception. 2017 Dec 20;8(6):2041669517747891. doi: 10.1177/2041669517747891 (PMC5761921; doi:10.1177/2041669517747891)
Supplement: Supplementary material [file Demonstration_of_Peripheral_Flicker_Illusion.pptx]

## Slide 1
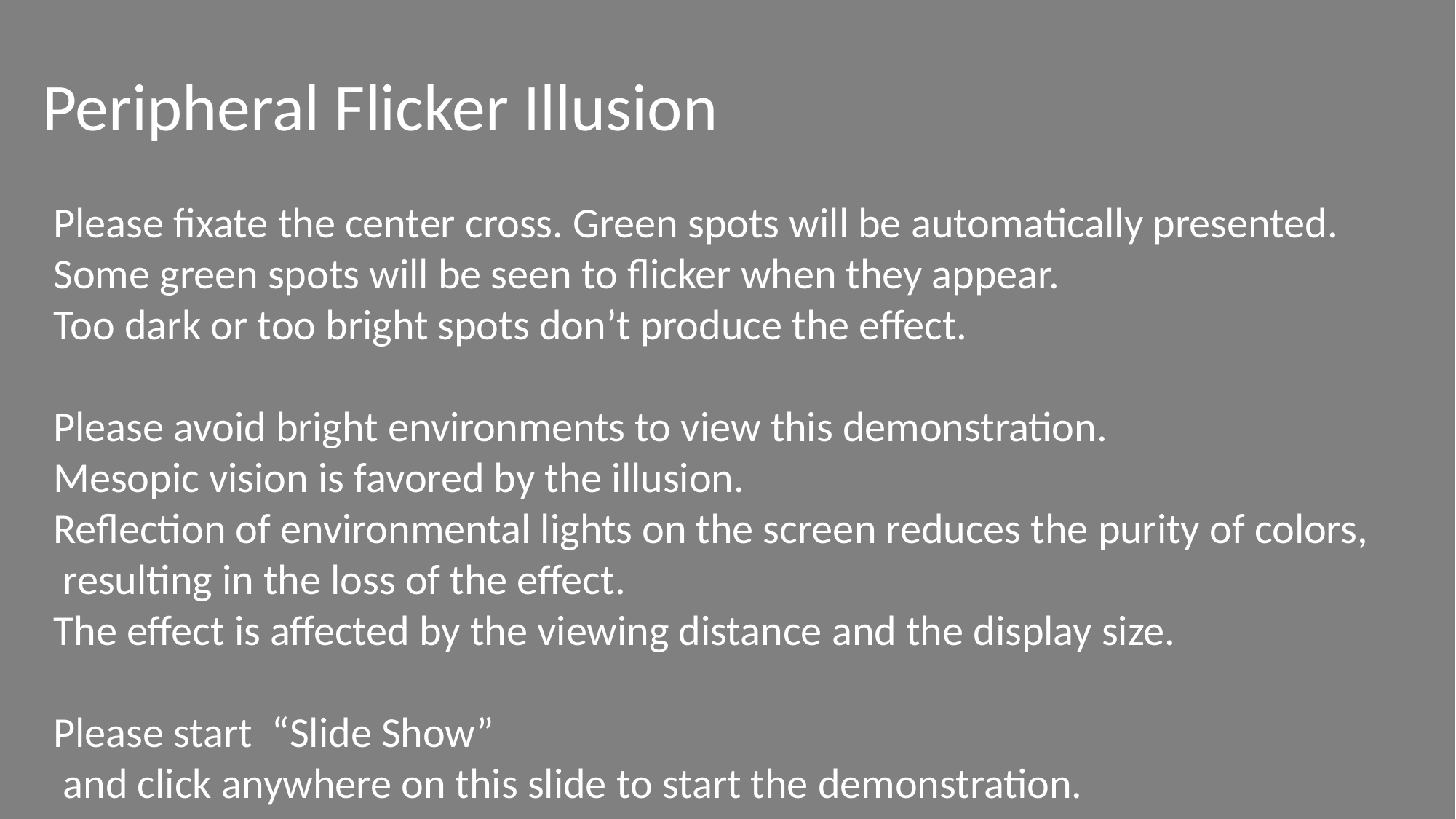

Peripheral Flicker Illusion
Please fixate the center cross. Green spots will be automatically presented.
Some green spots will be seen to flicker when they appear.
Too dark or too bright spots don’t produce the effect.
Please avoid bright environments to view this demonstration.
Mesopic vision is favored by the illusion.
Reflection of environmental lights on the screen reduces the purity of colors,
 resulting in the loss of the effect.
The effect is affected by the viewing distance and the display size.
Please start “Slide Show”
 and click anywhere on this slide to start the demonstration.

## Slide 2
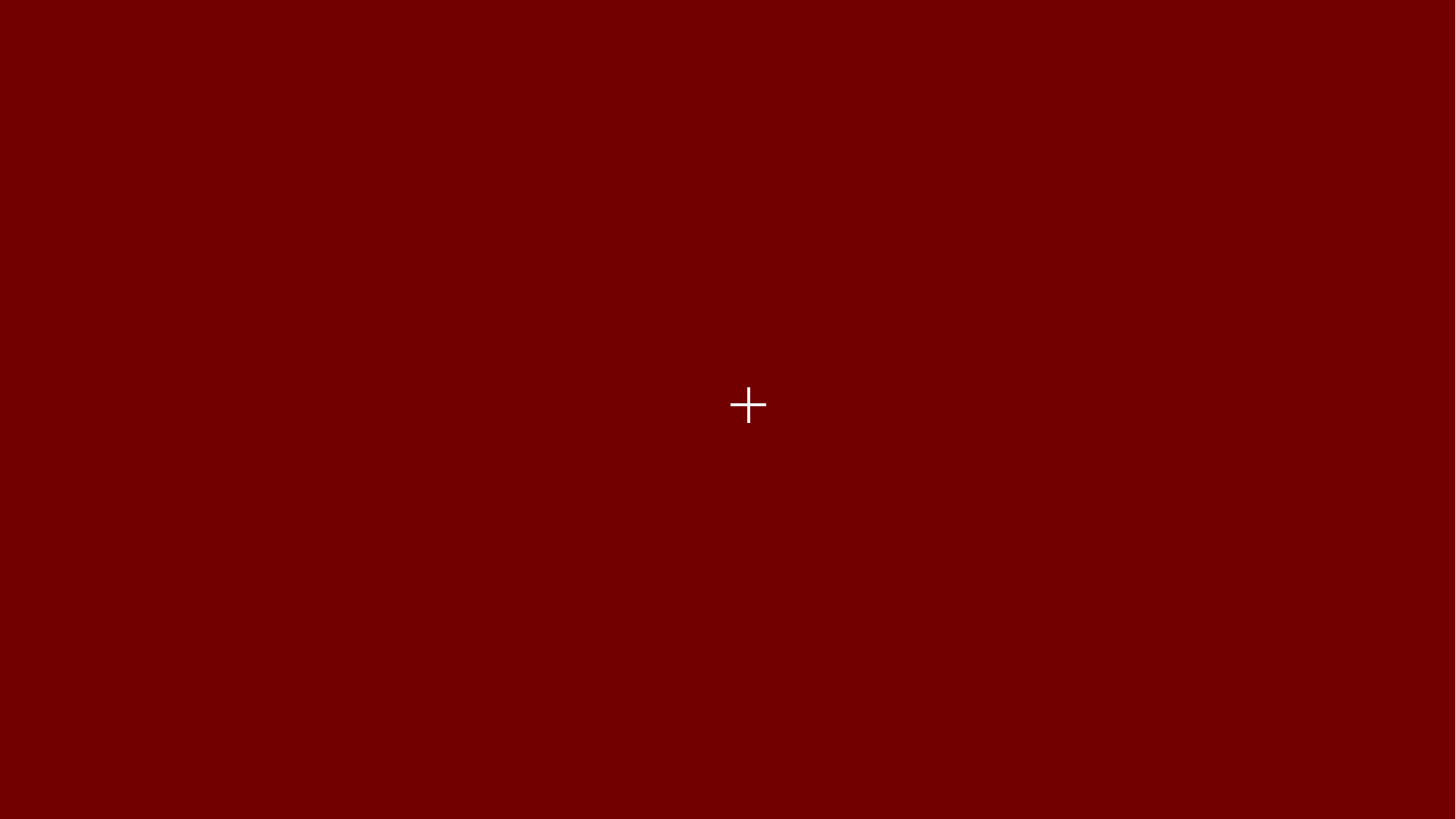

## Slide 3
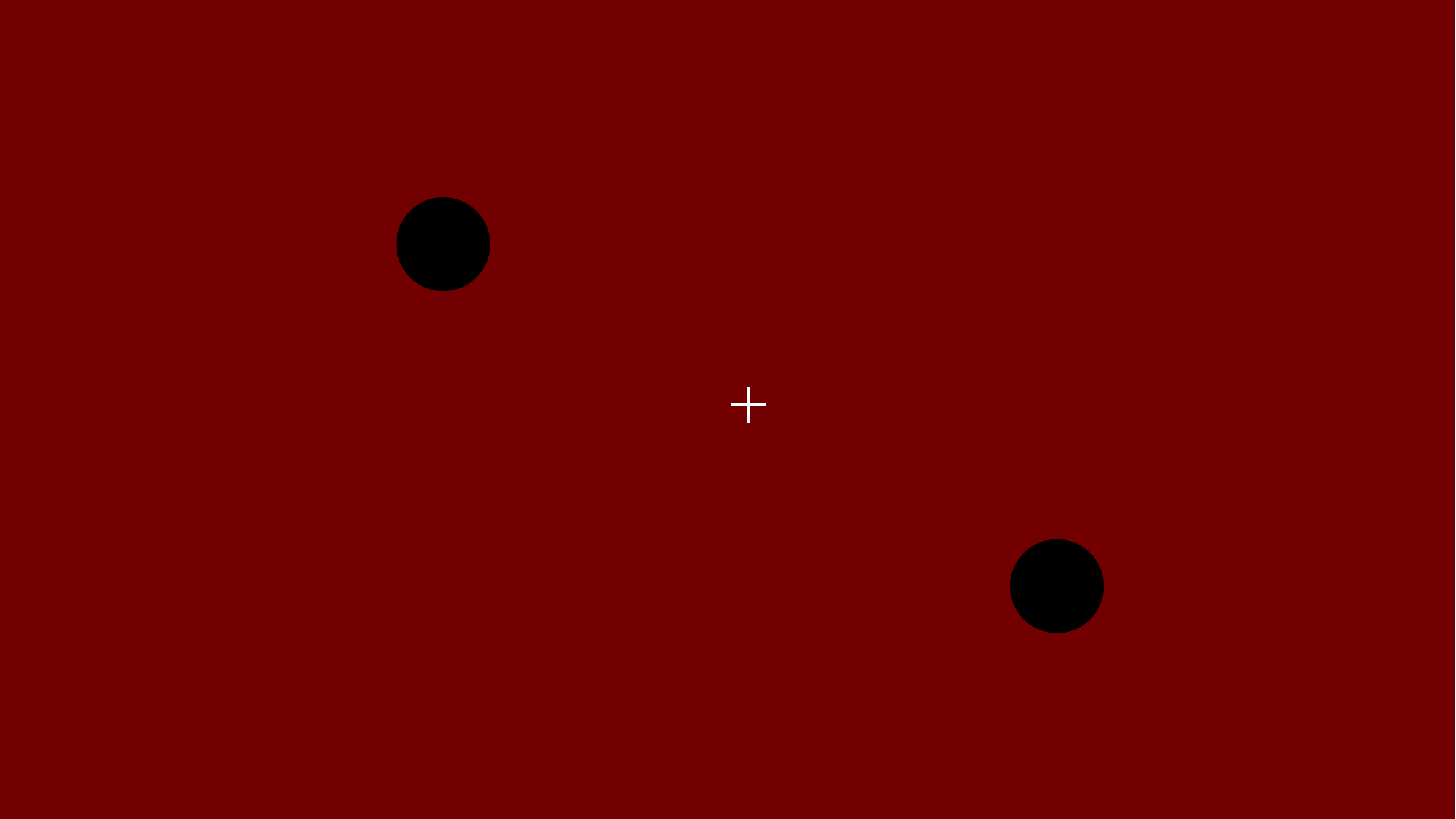

## Slide 4
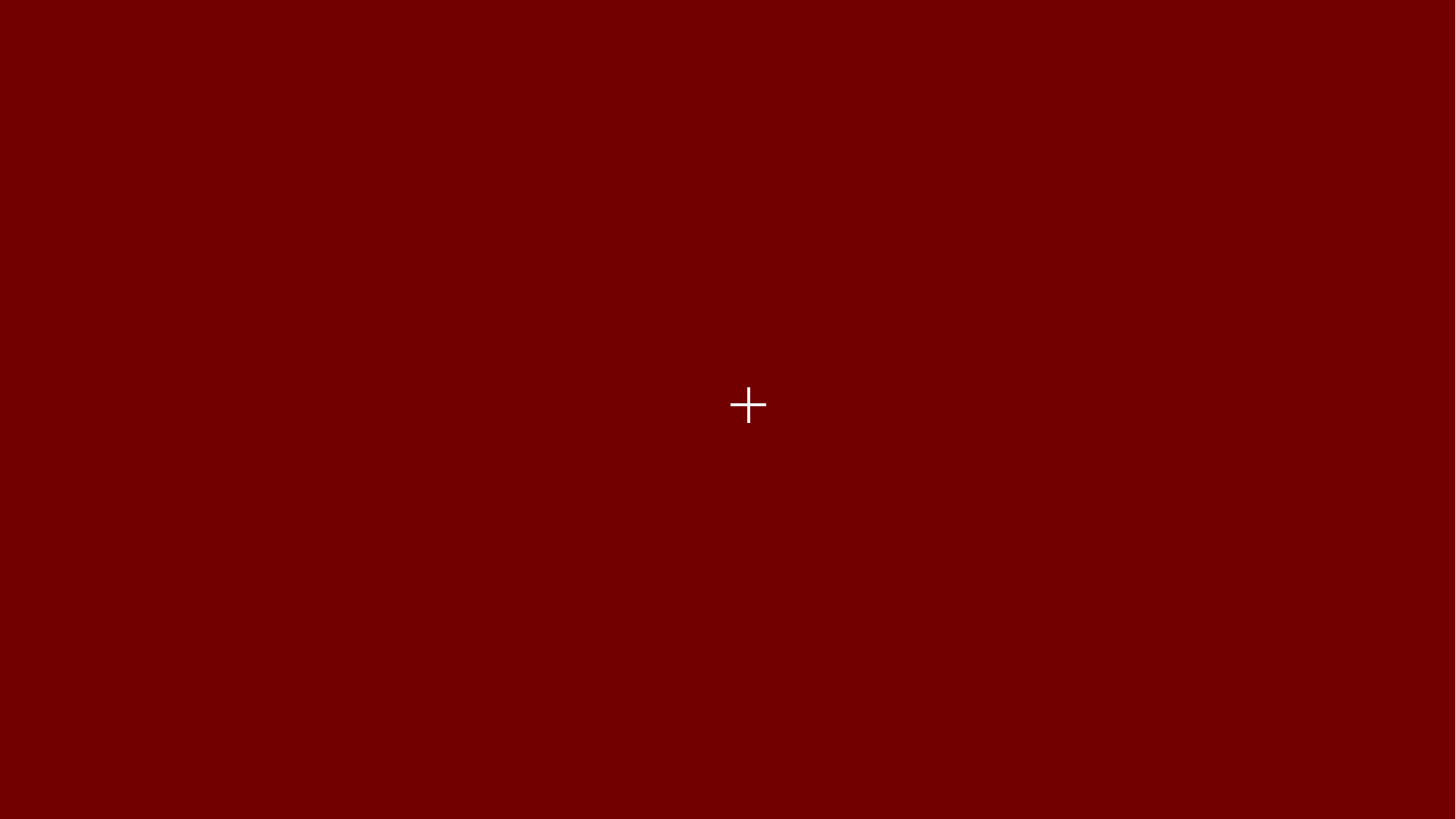

## Slide 5
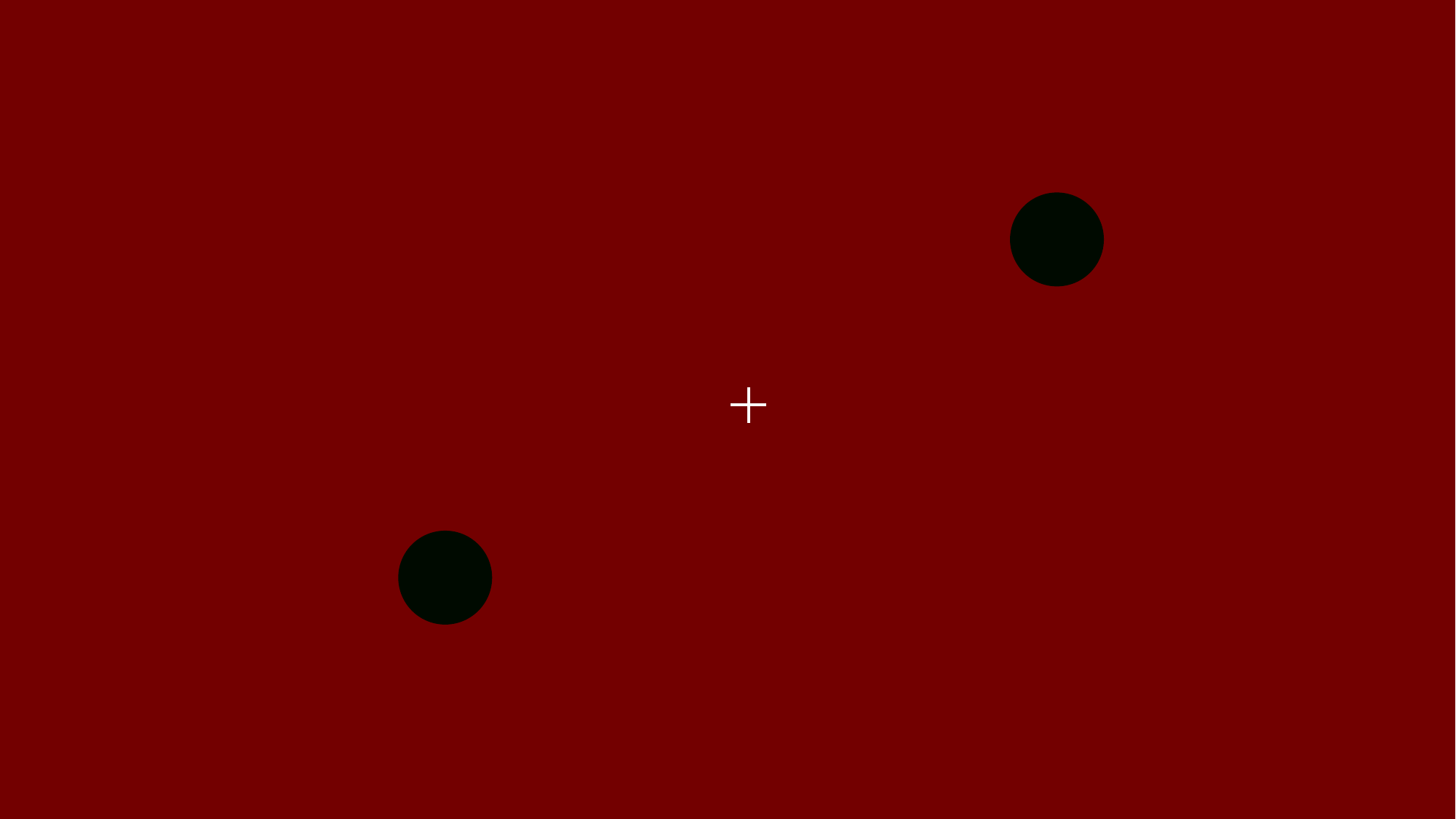

## Slide 6
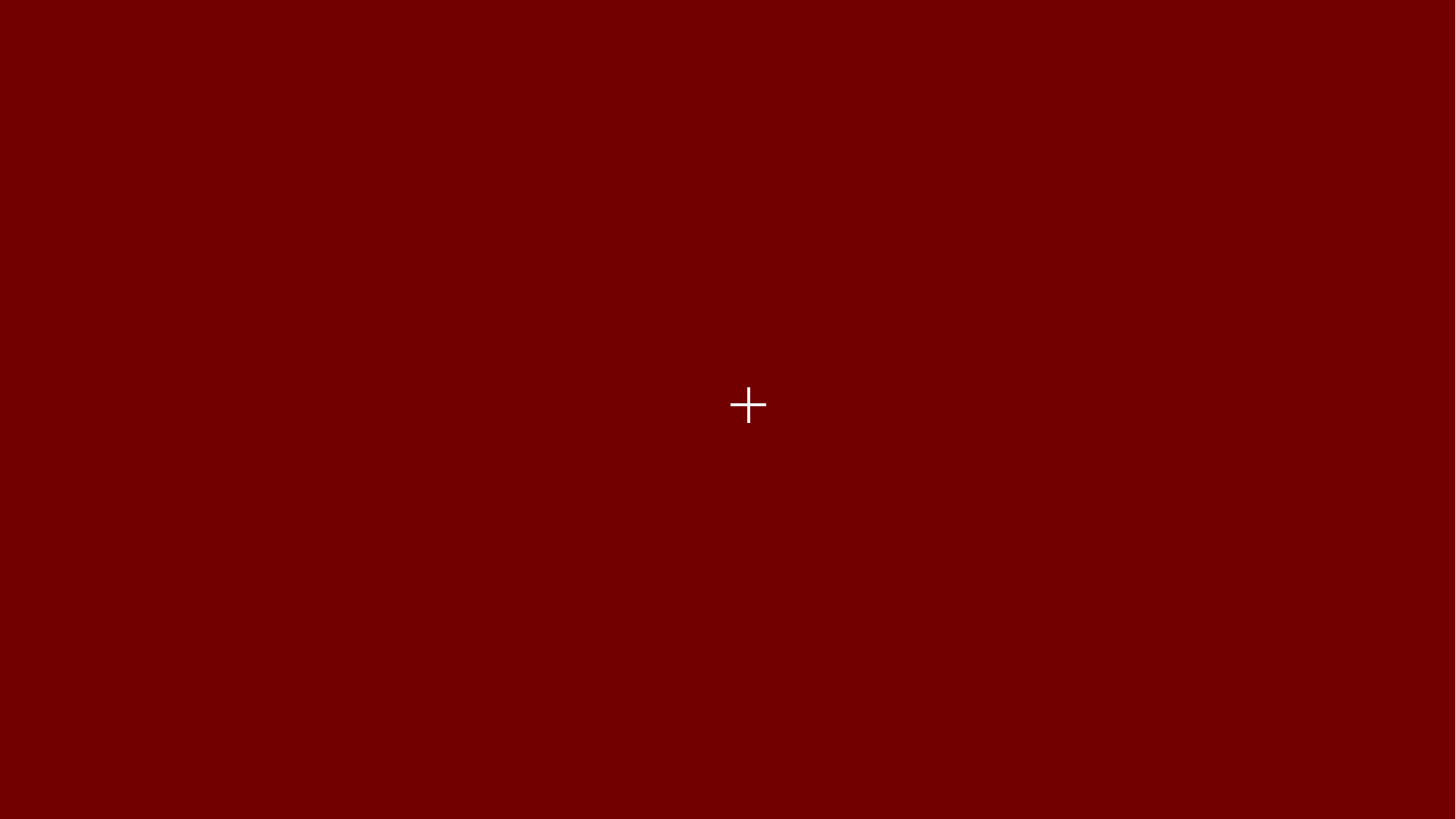

## Slide 7
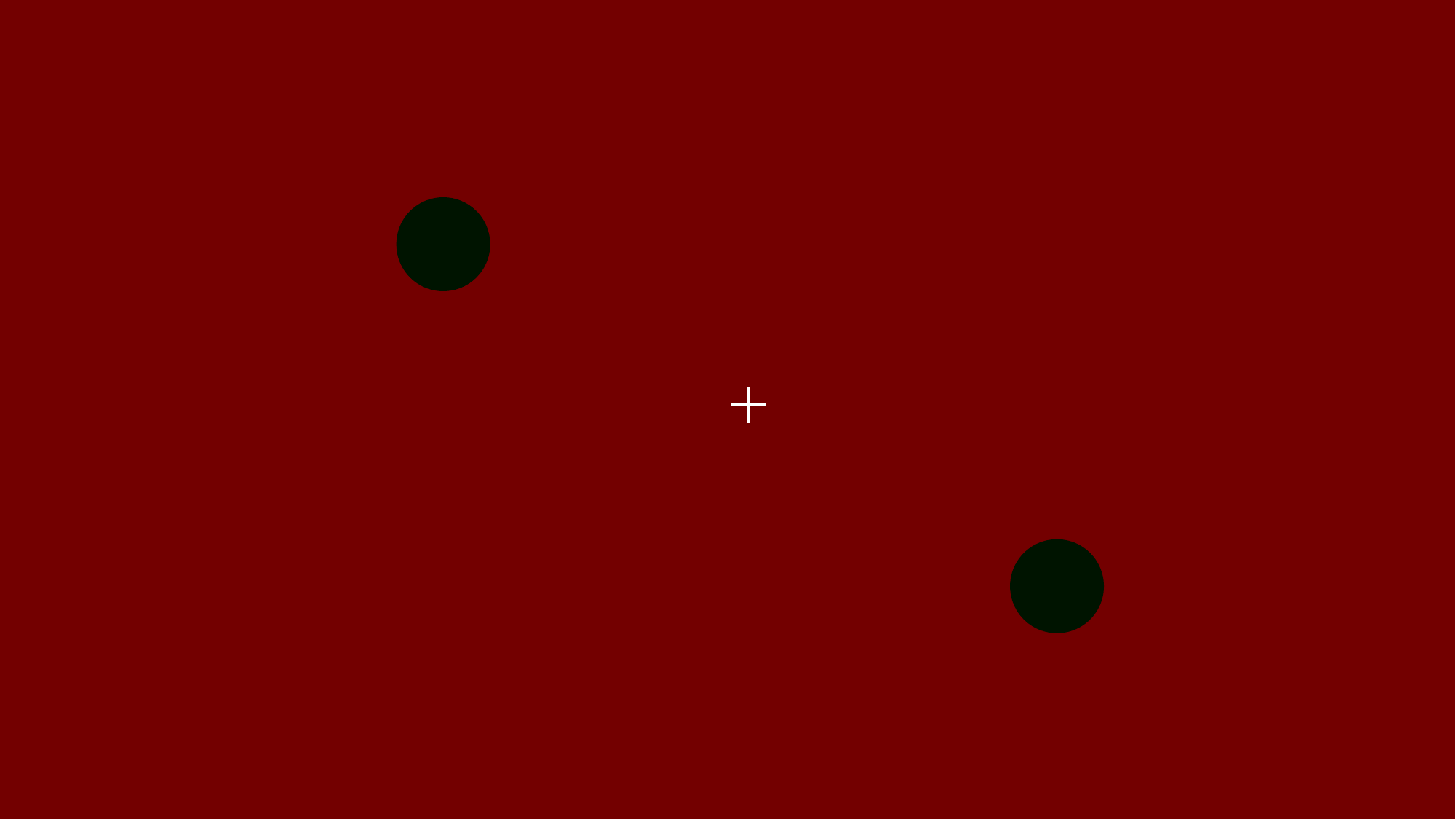

## Slide 8
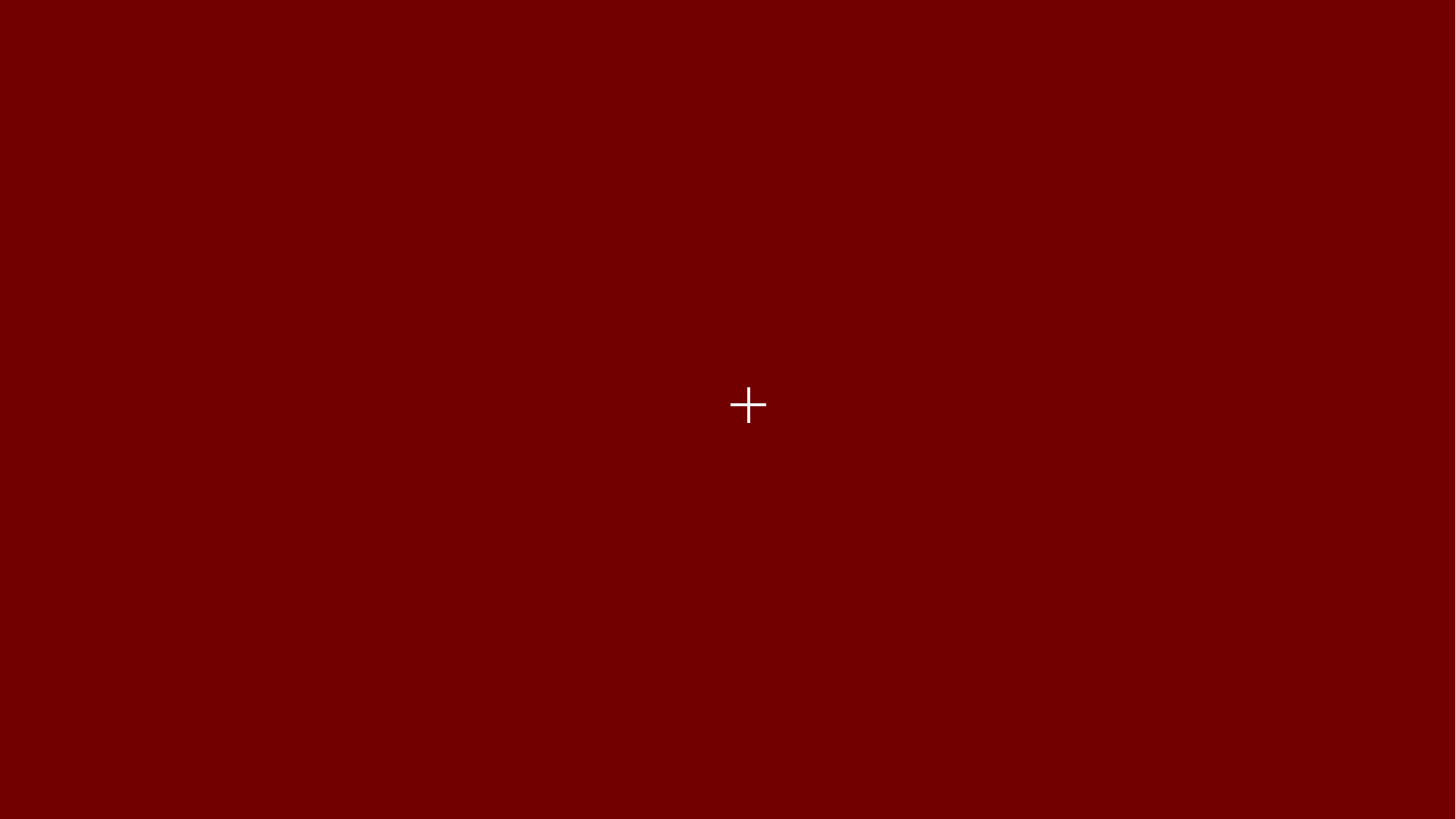

## Slide 9
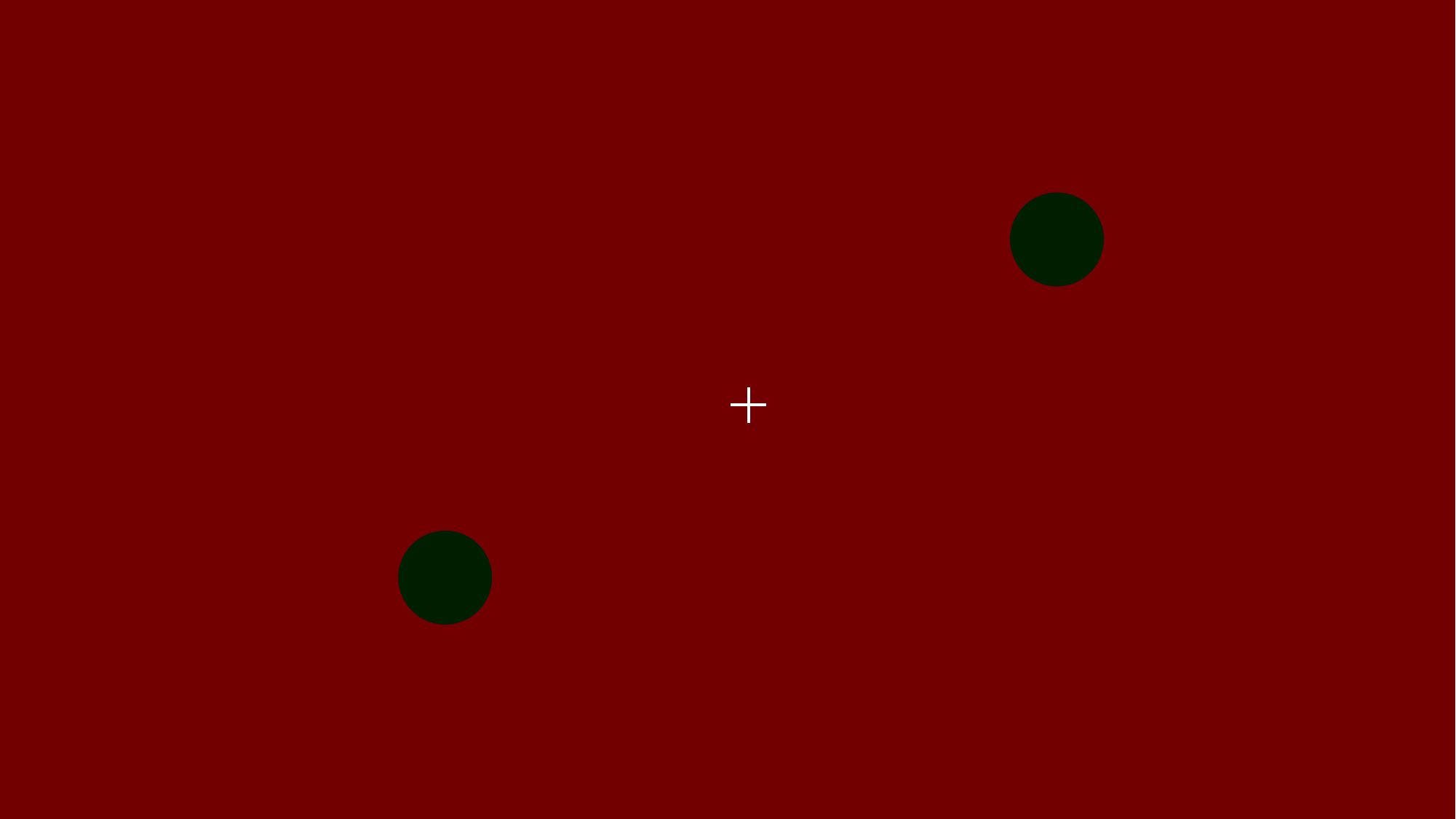

## Slide 10
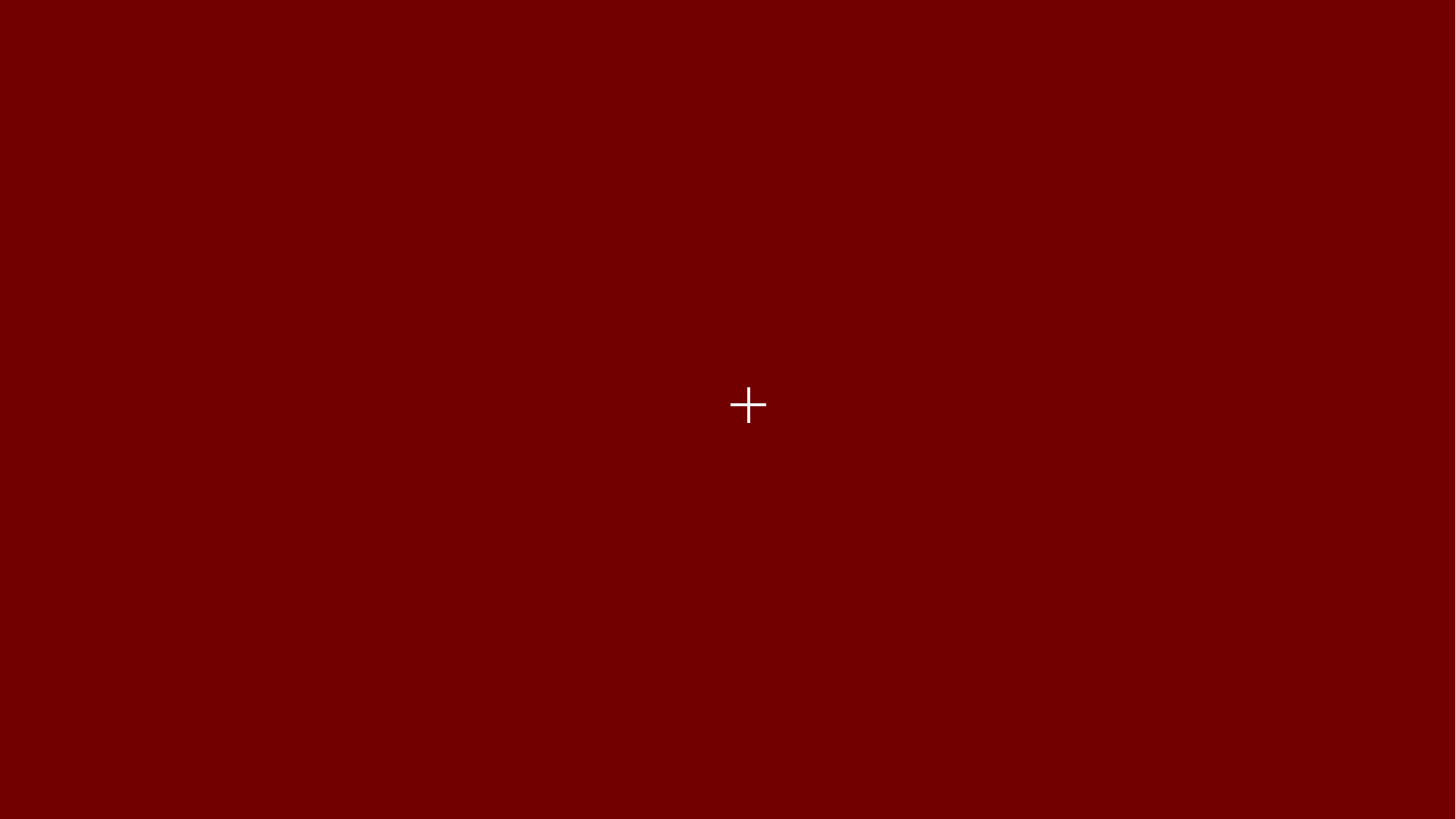

## Slide 11
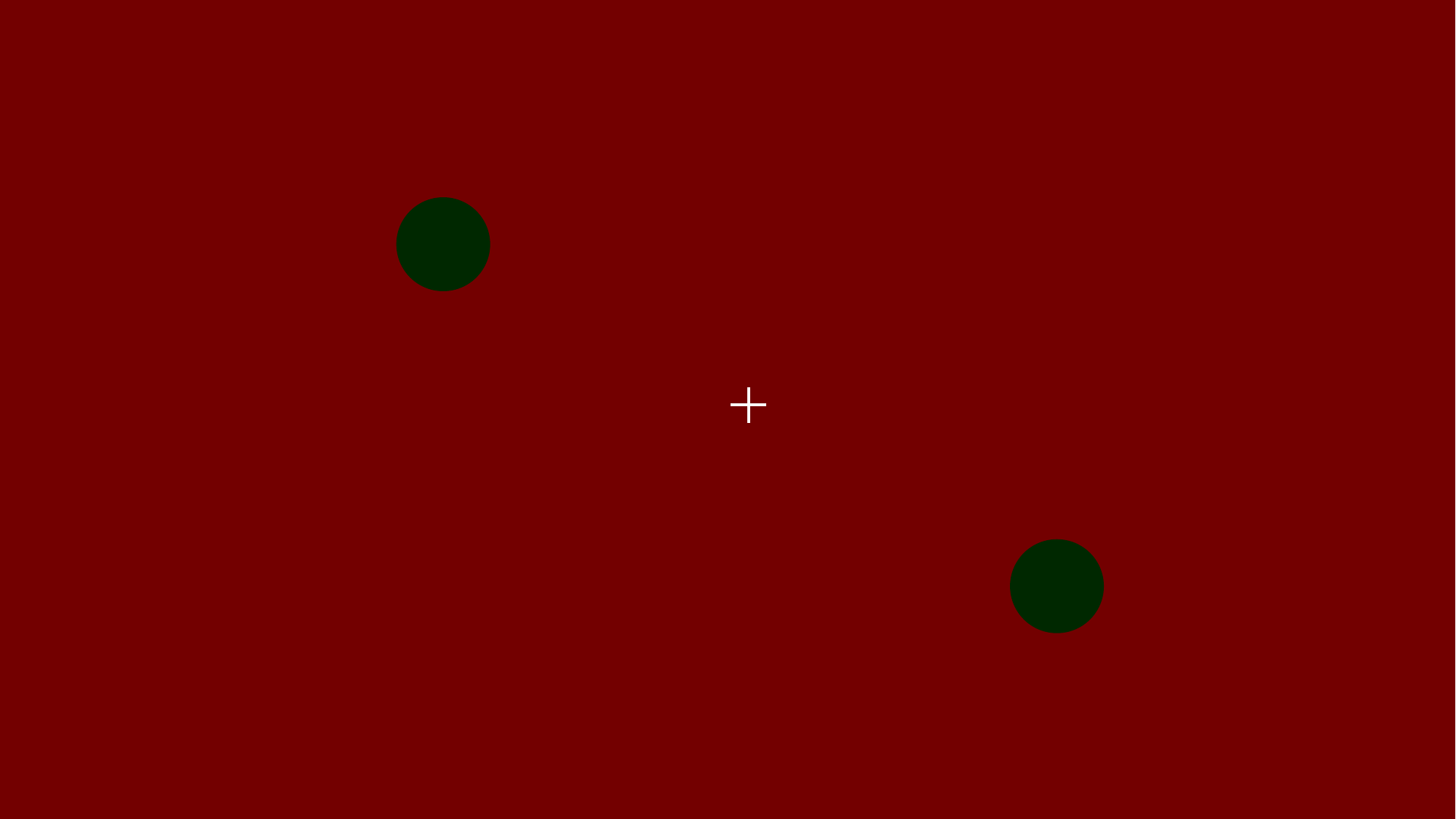

## Slide 12
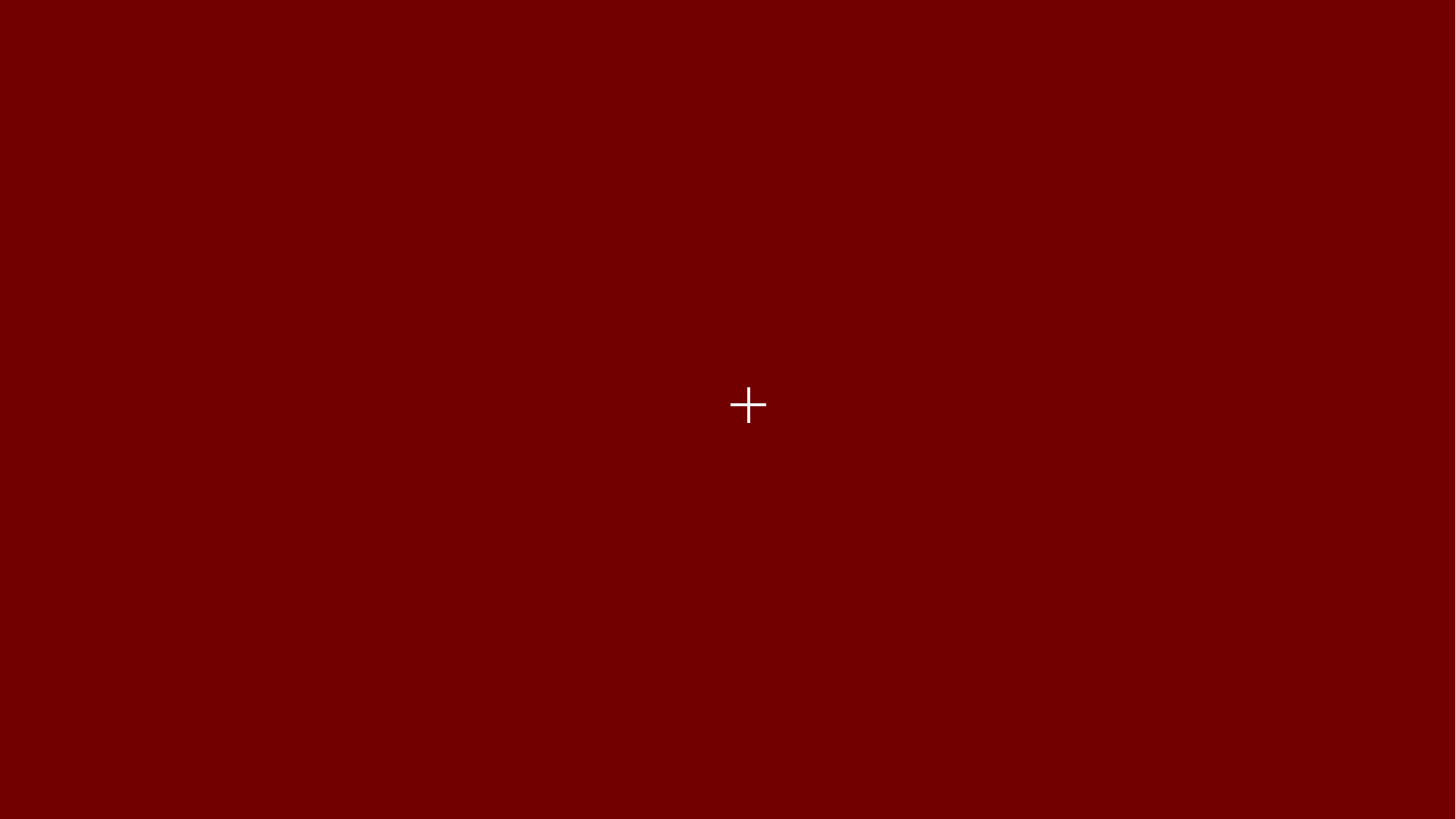

## Slide 13
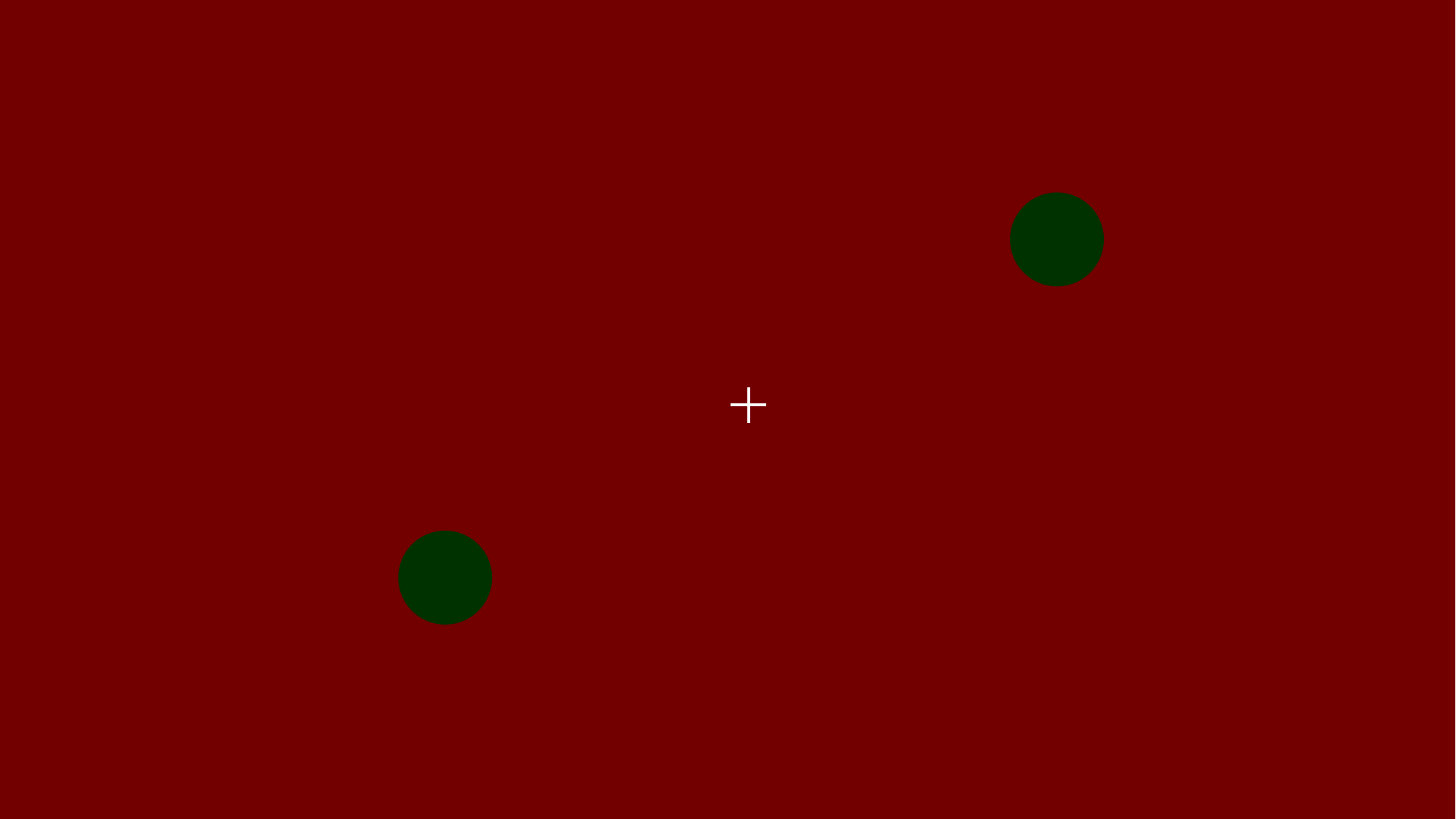

## Slide 14
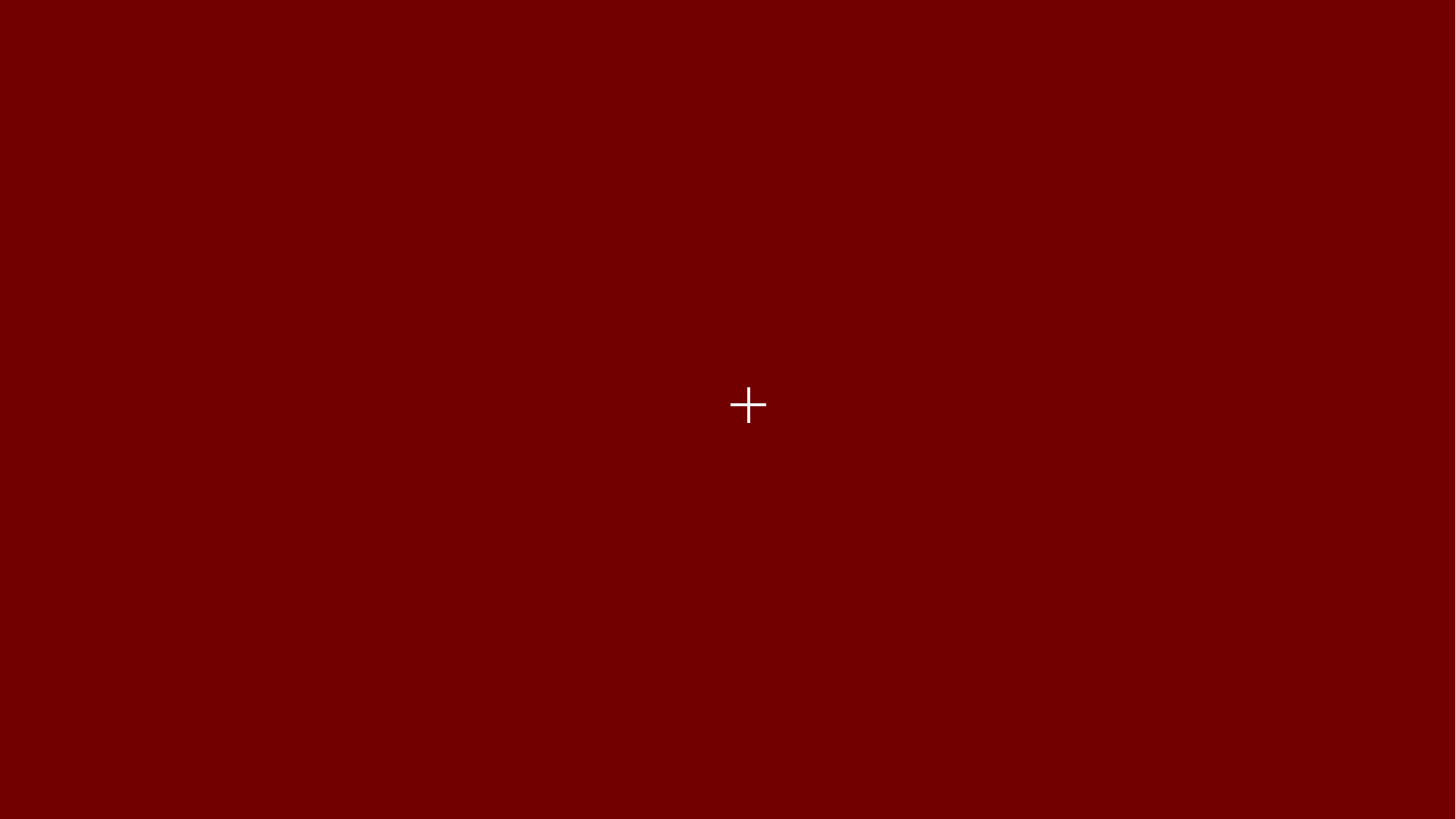

## Slide 15
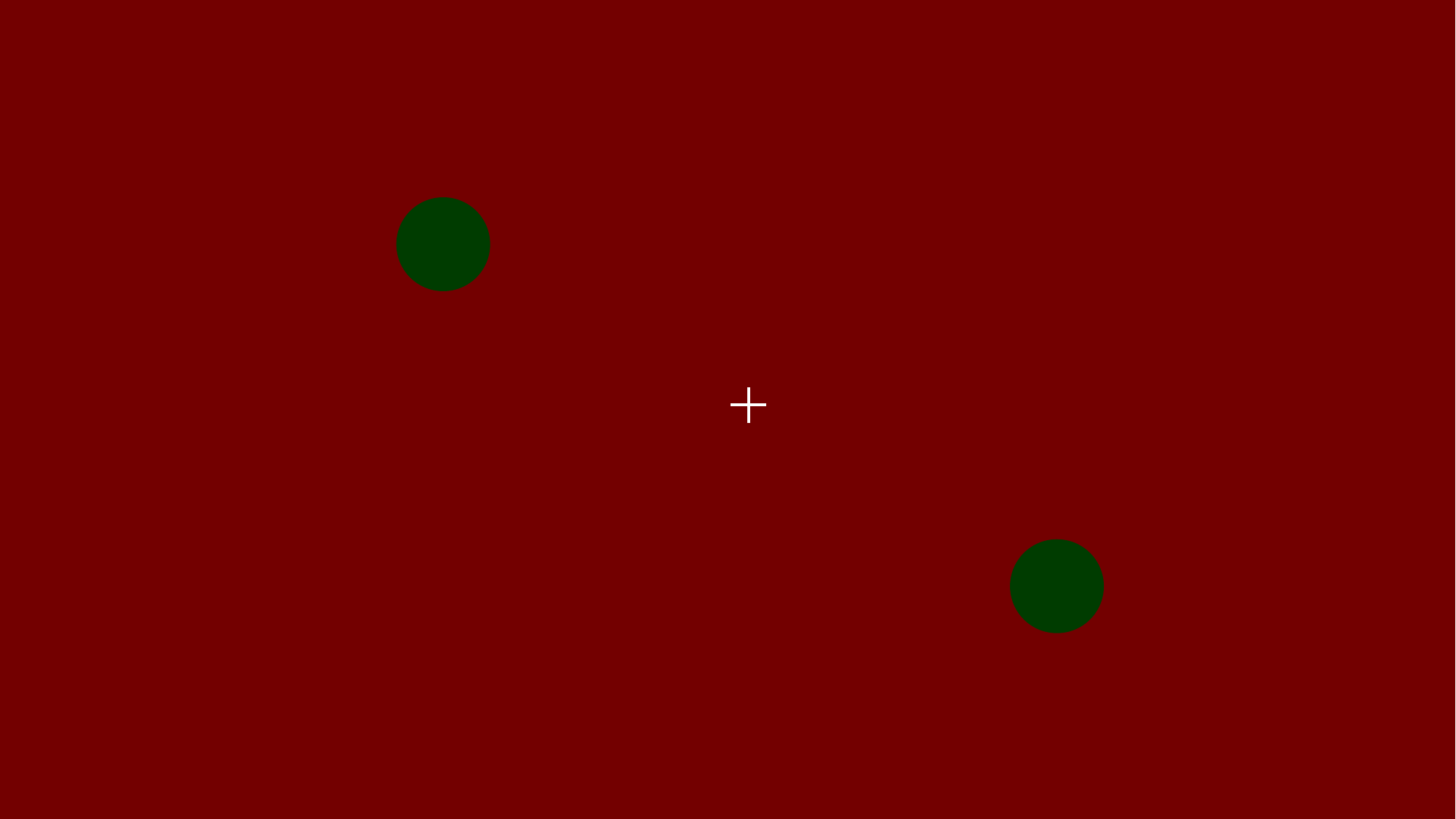

## Slide 16
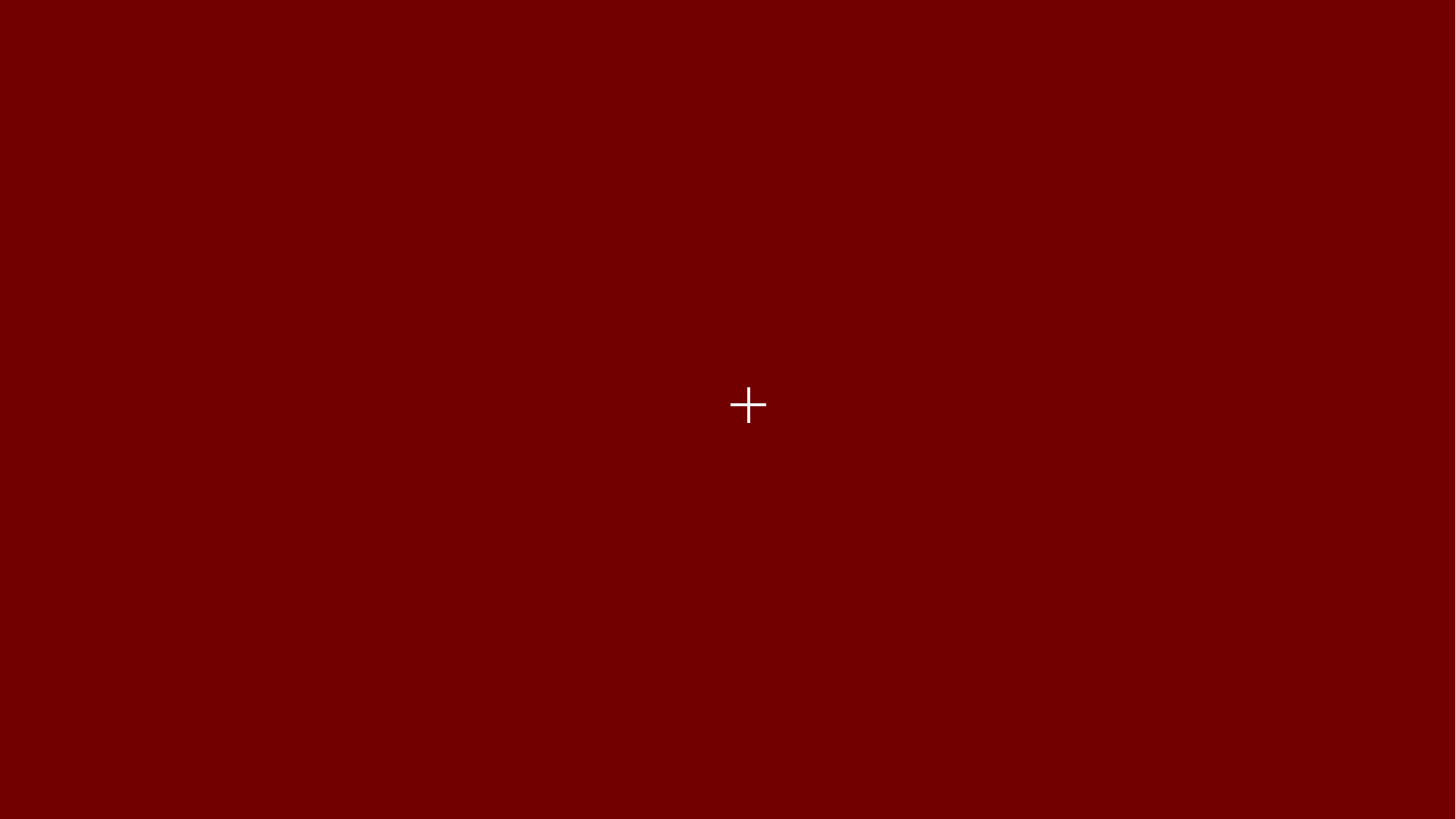

## Slide 17
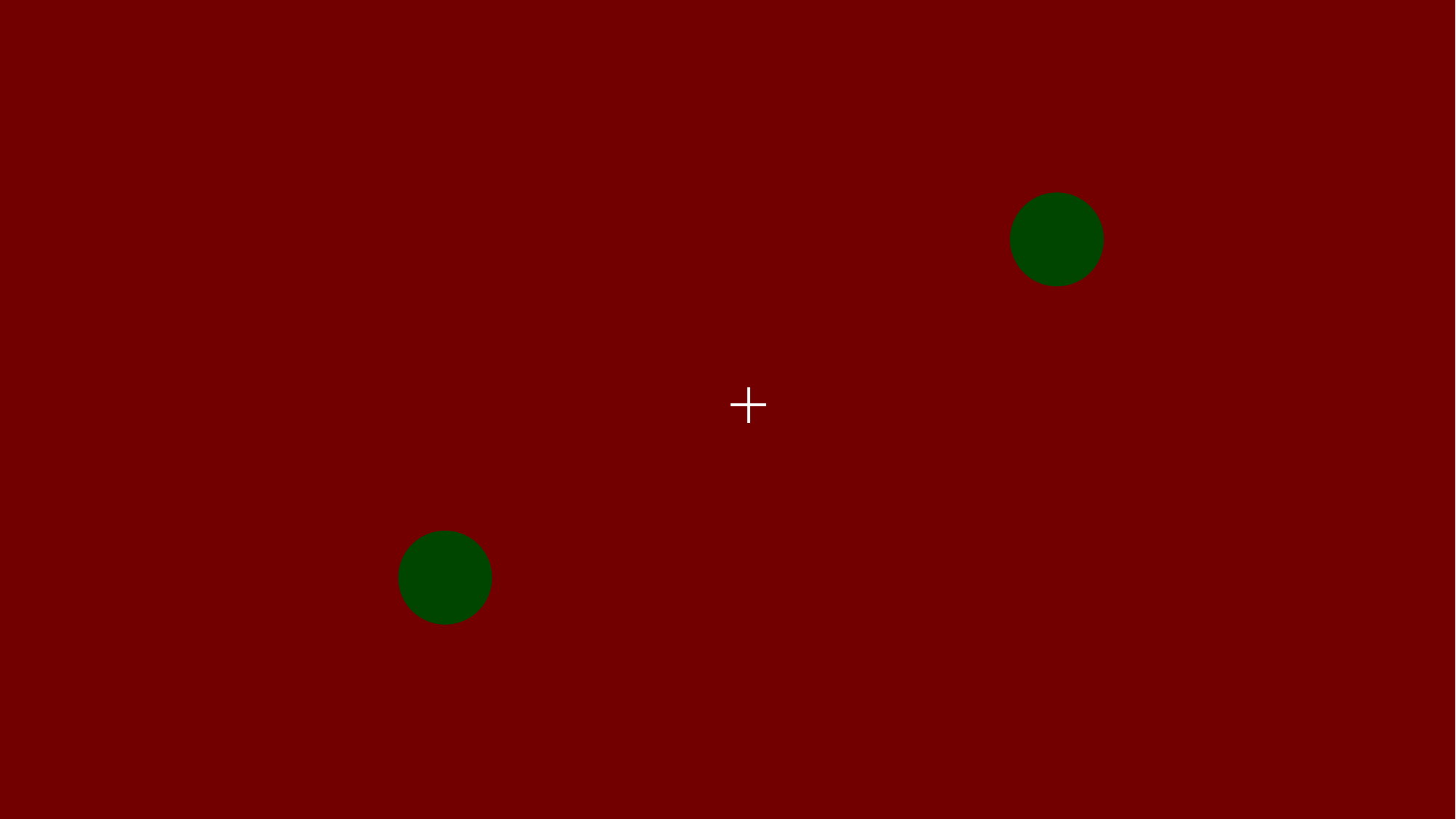

## Slide 18
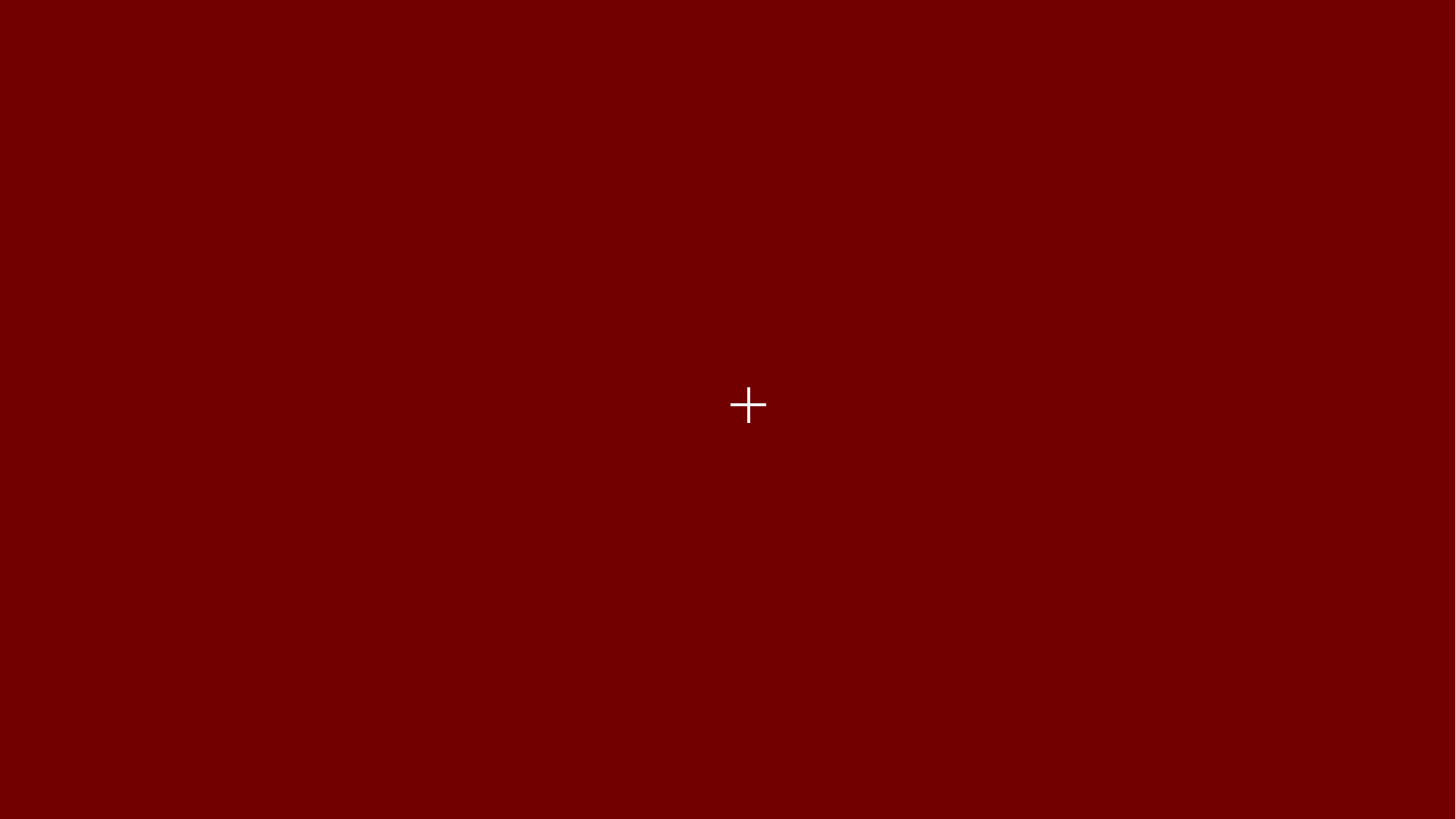

## Slide 19
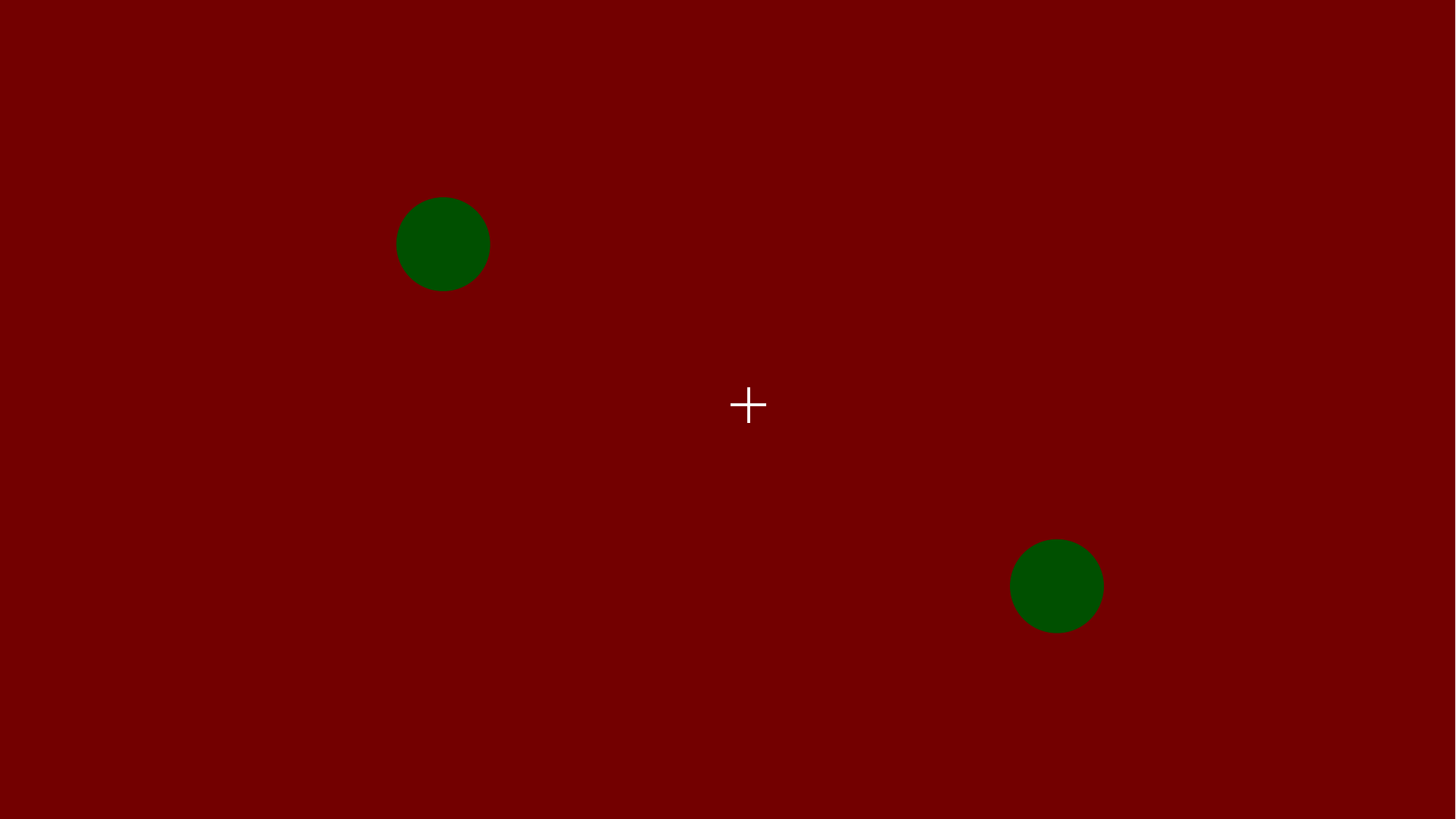

## Slide 20
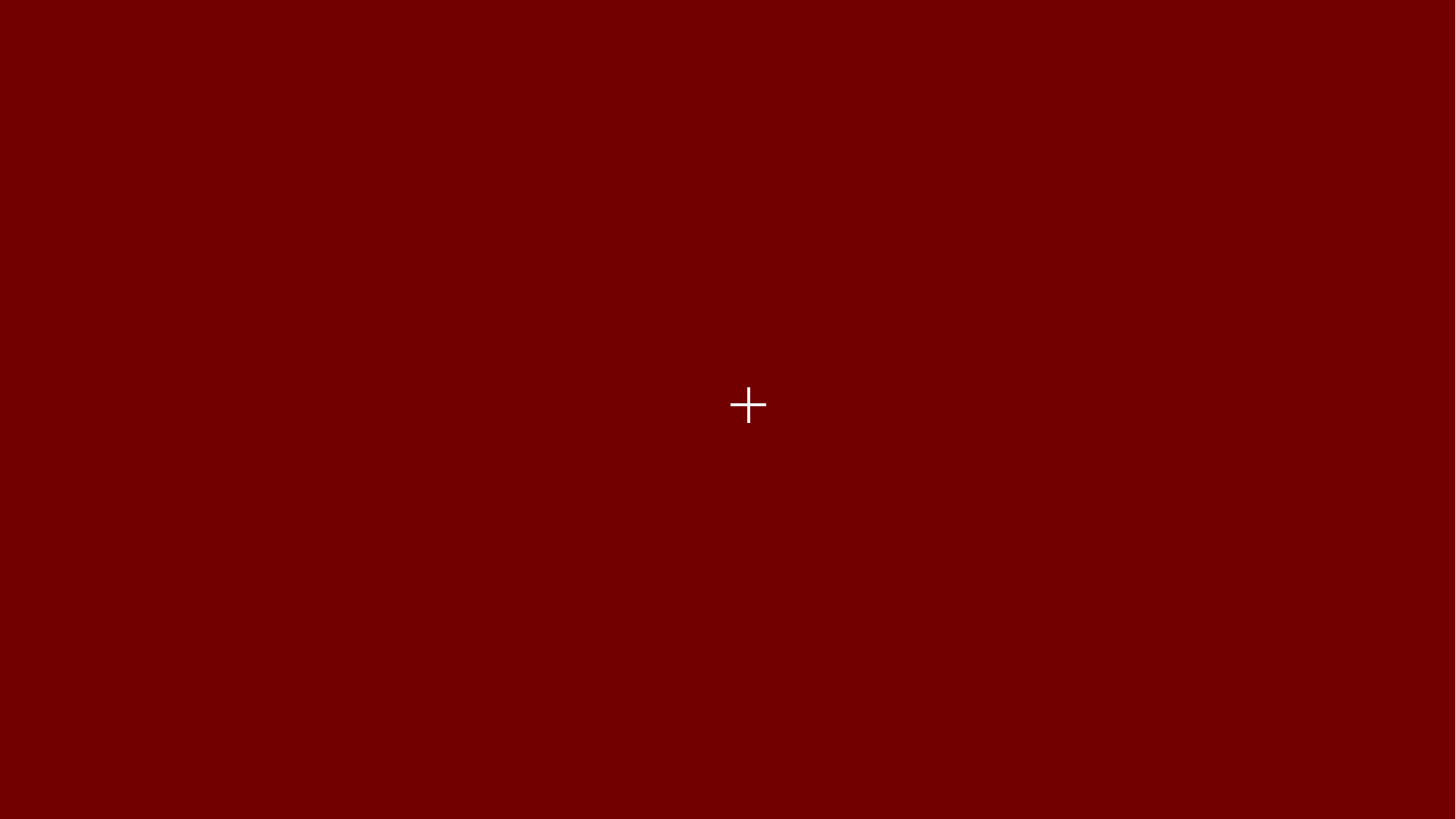

## Slide 21
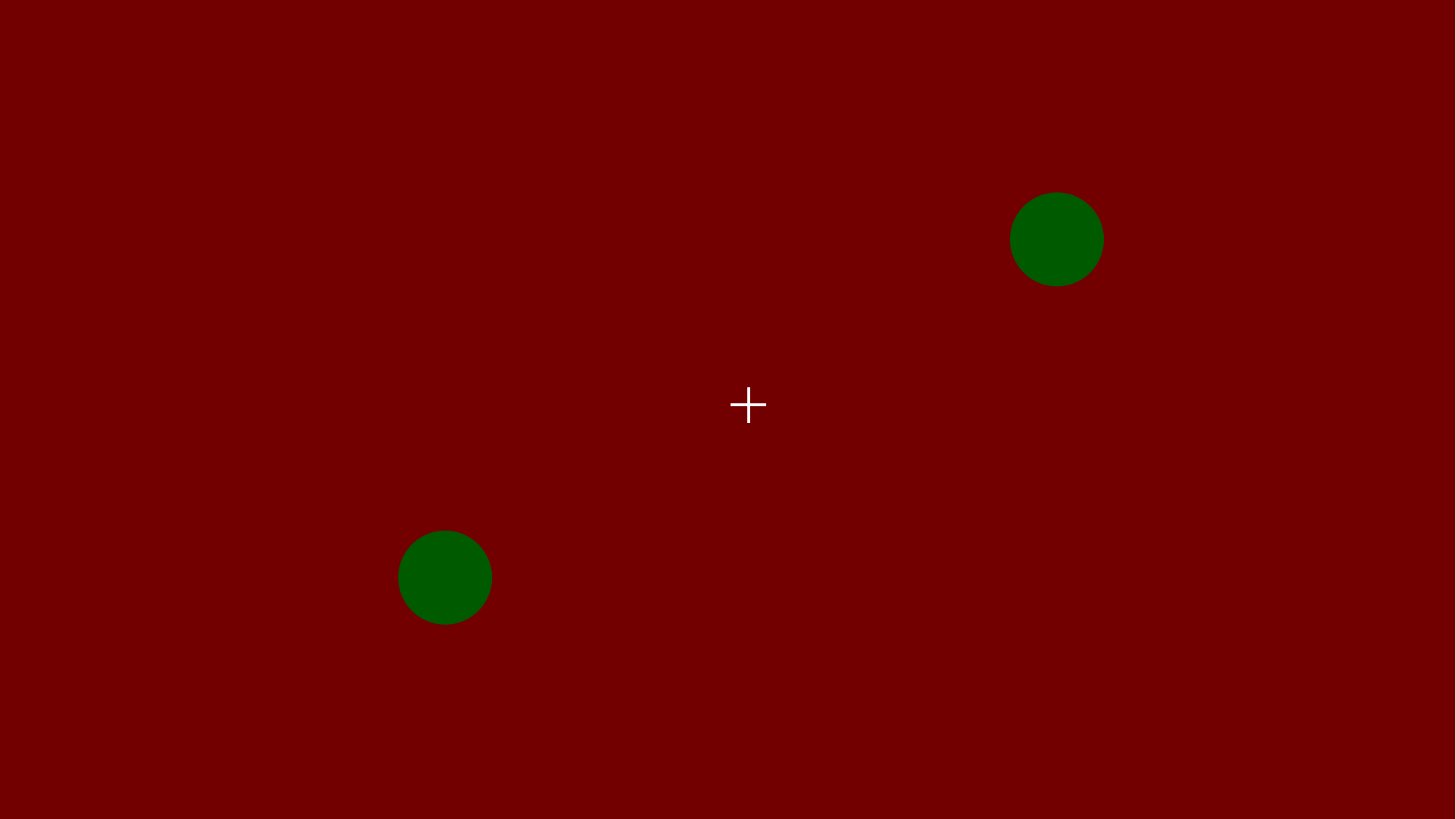

## Slide 22
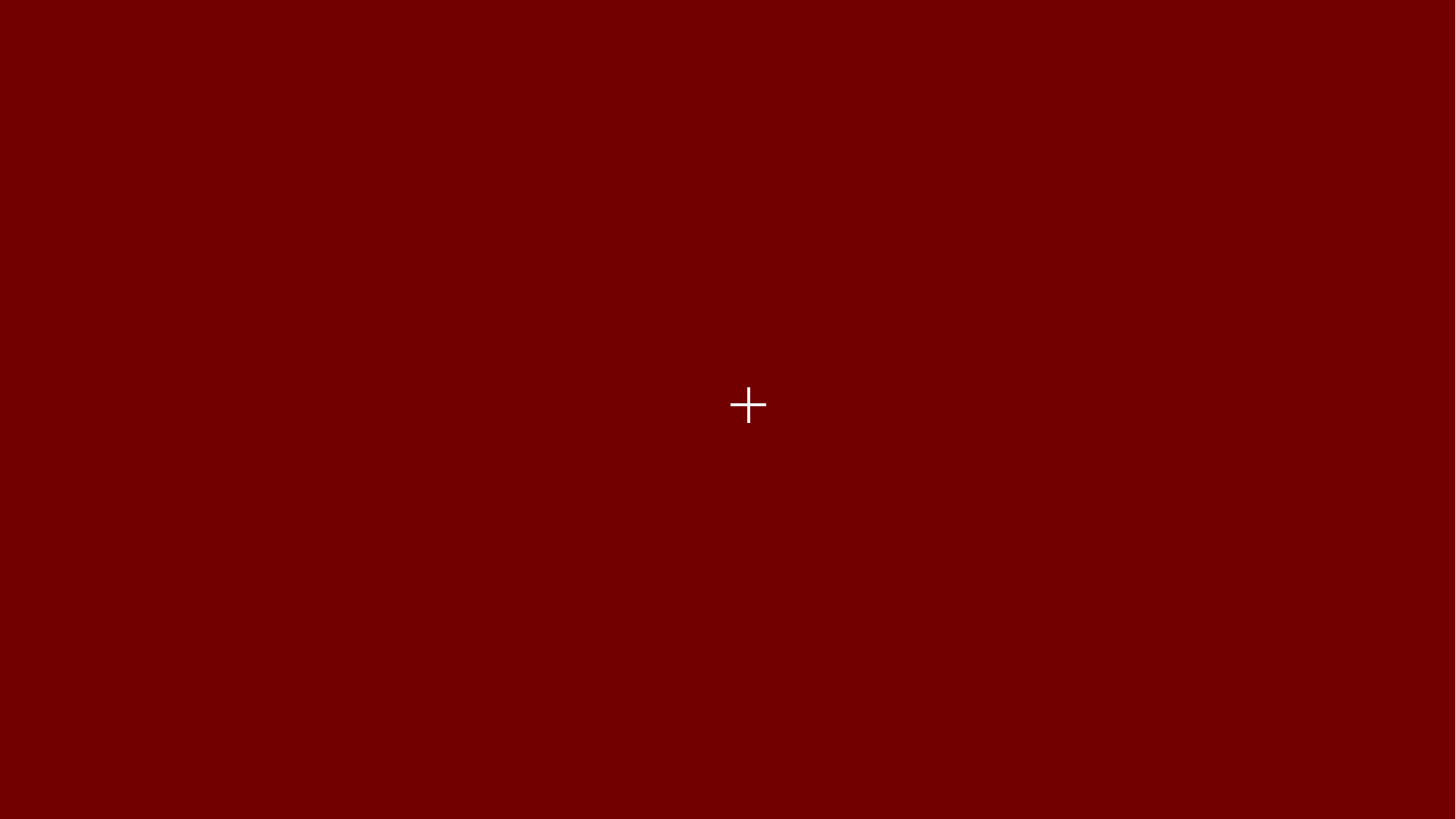

## Slide 23
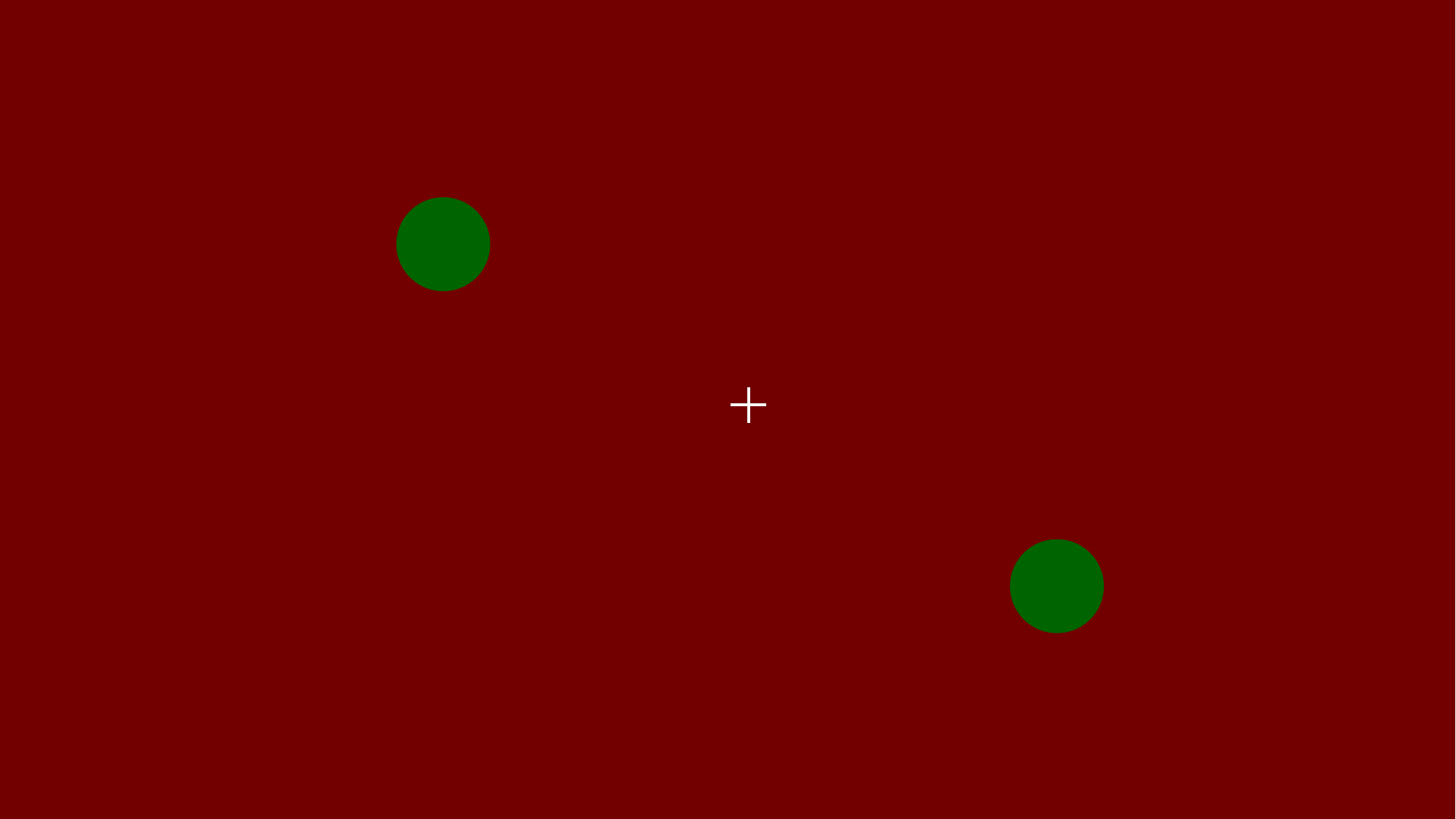

## Slide 24
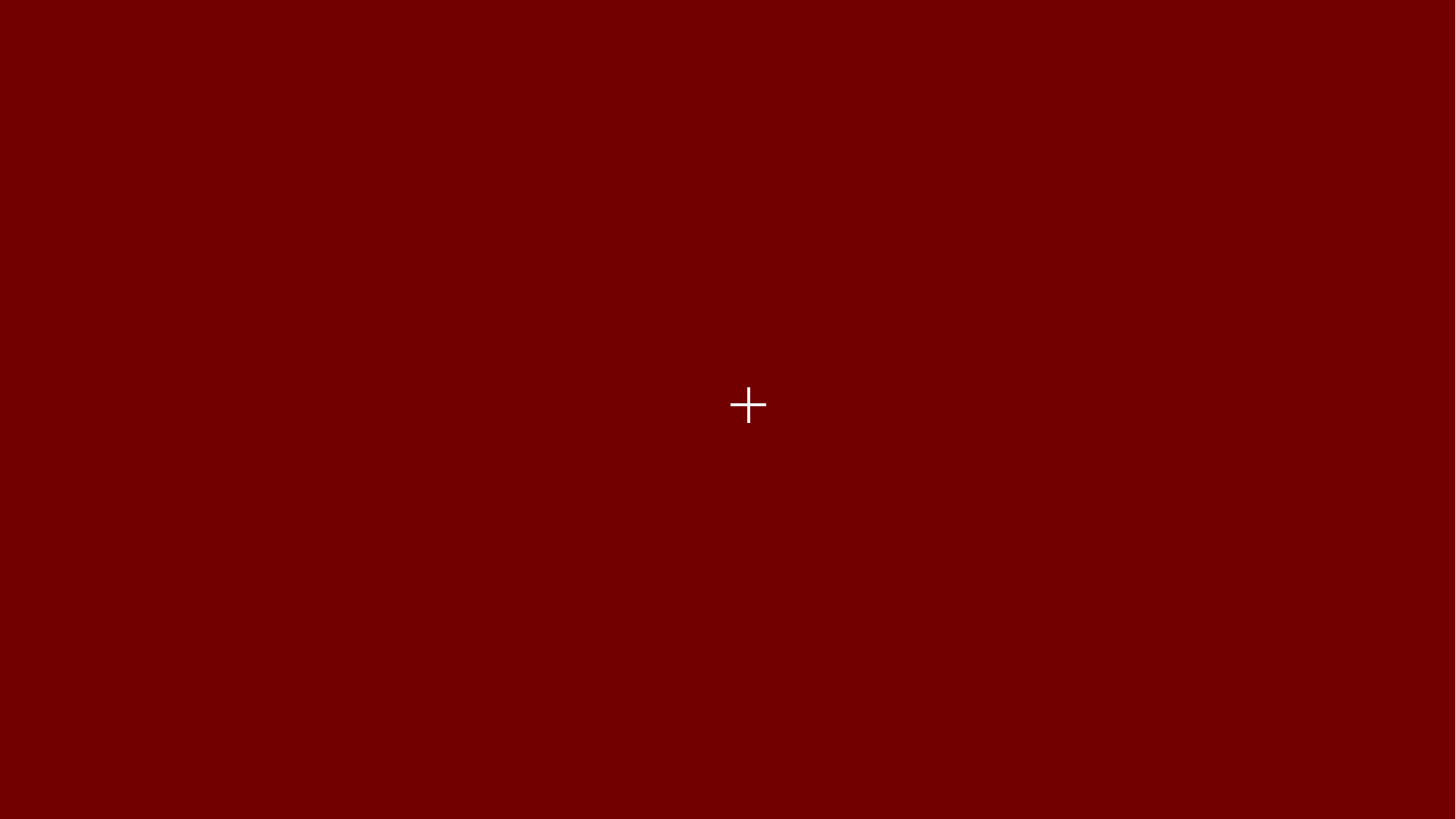

## Slide 25
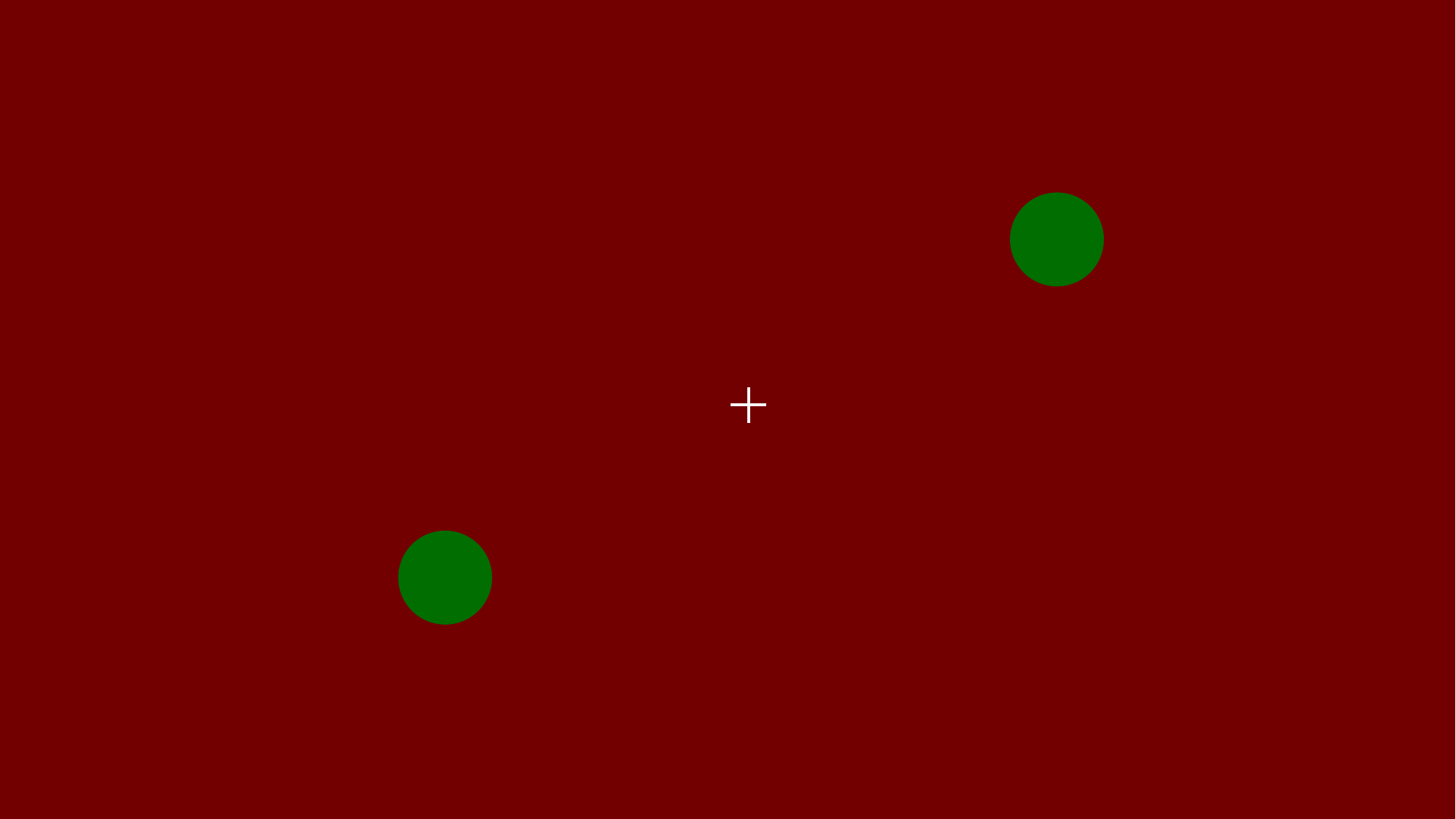

## Slide 26
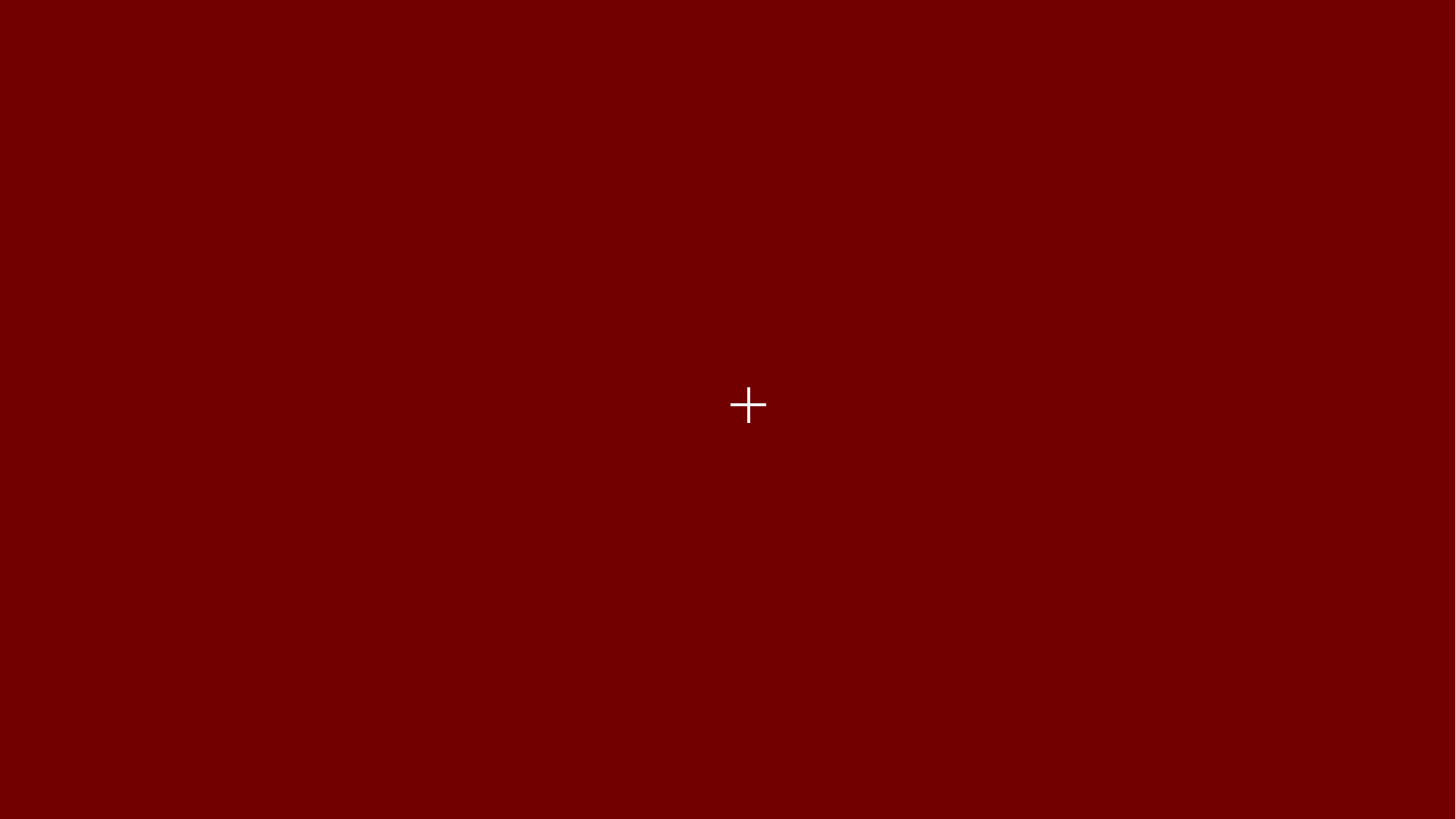

## Slide 27
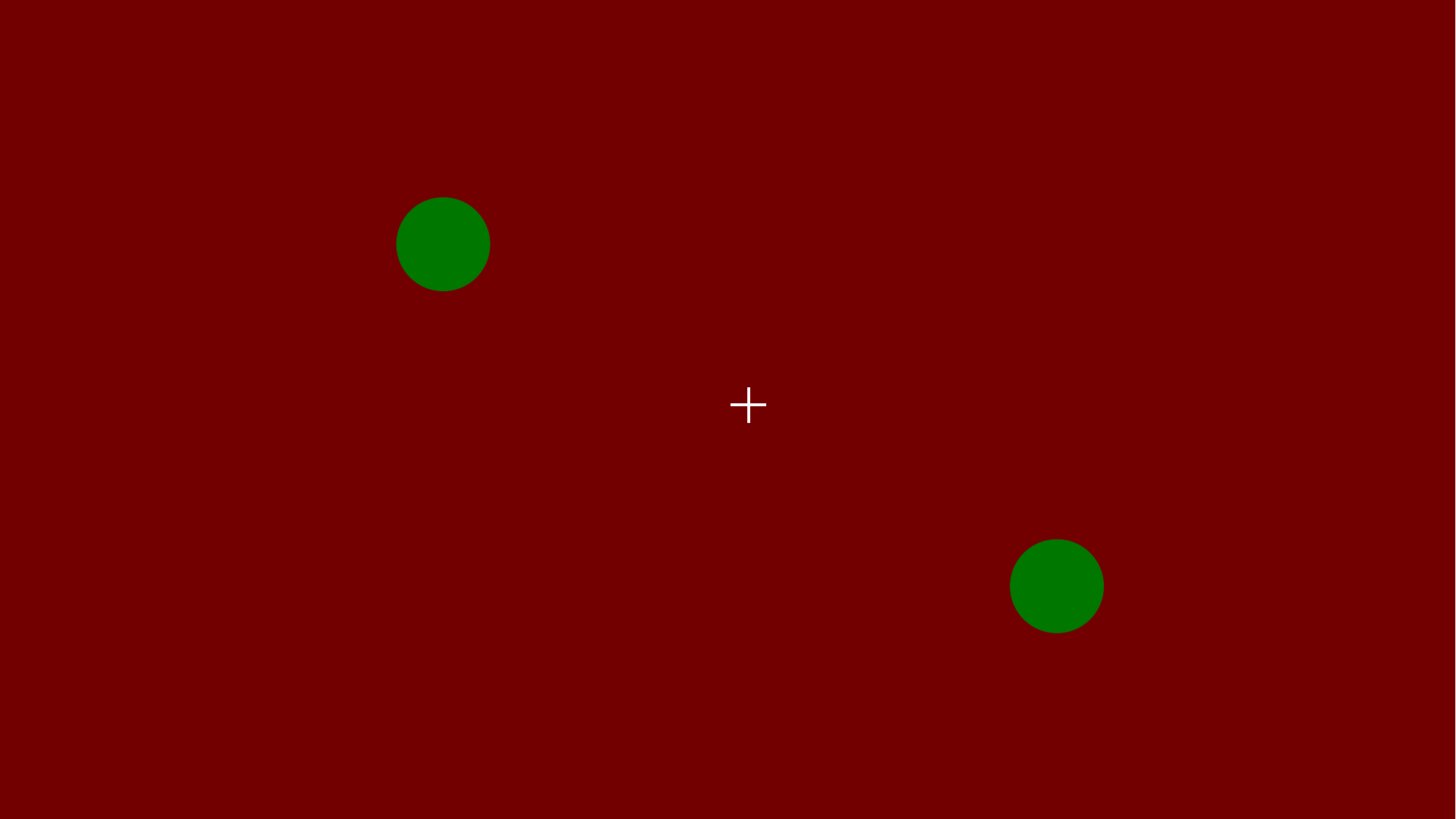

## Slide 28
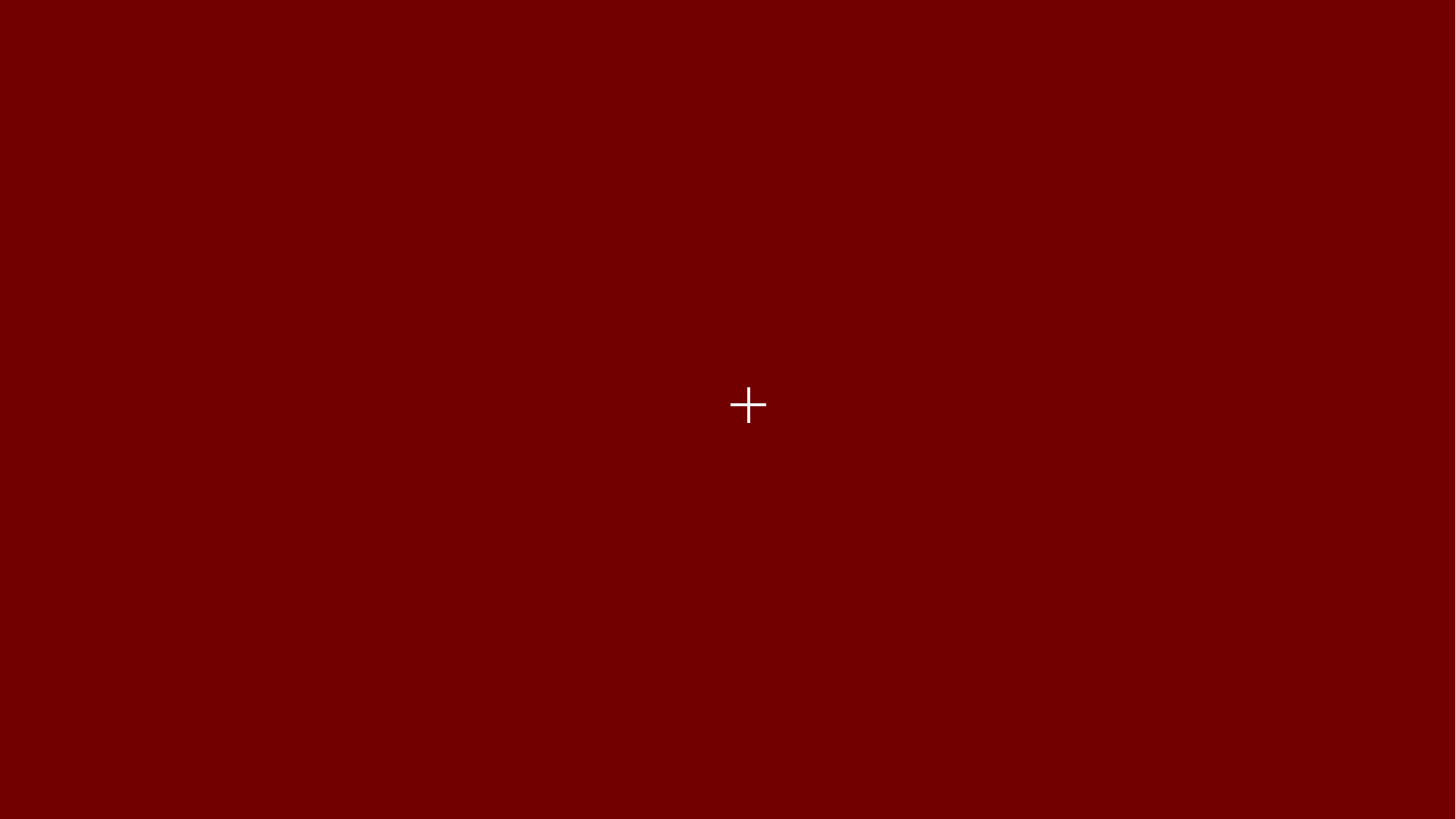

## Slide 29
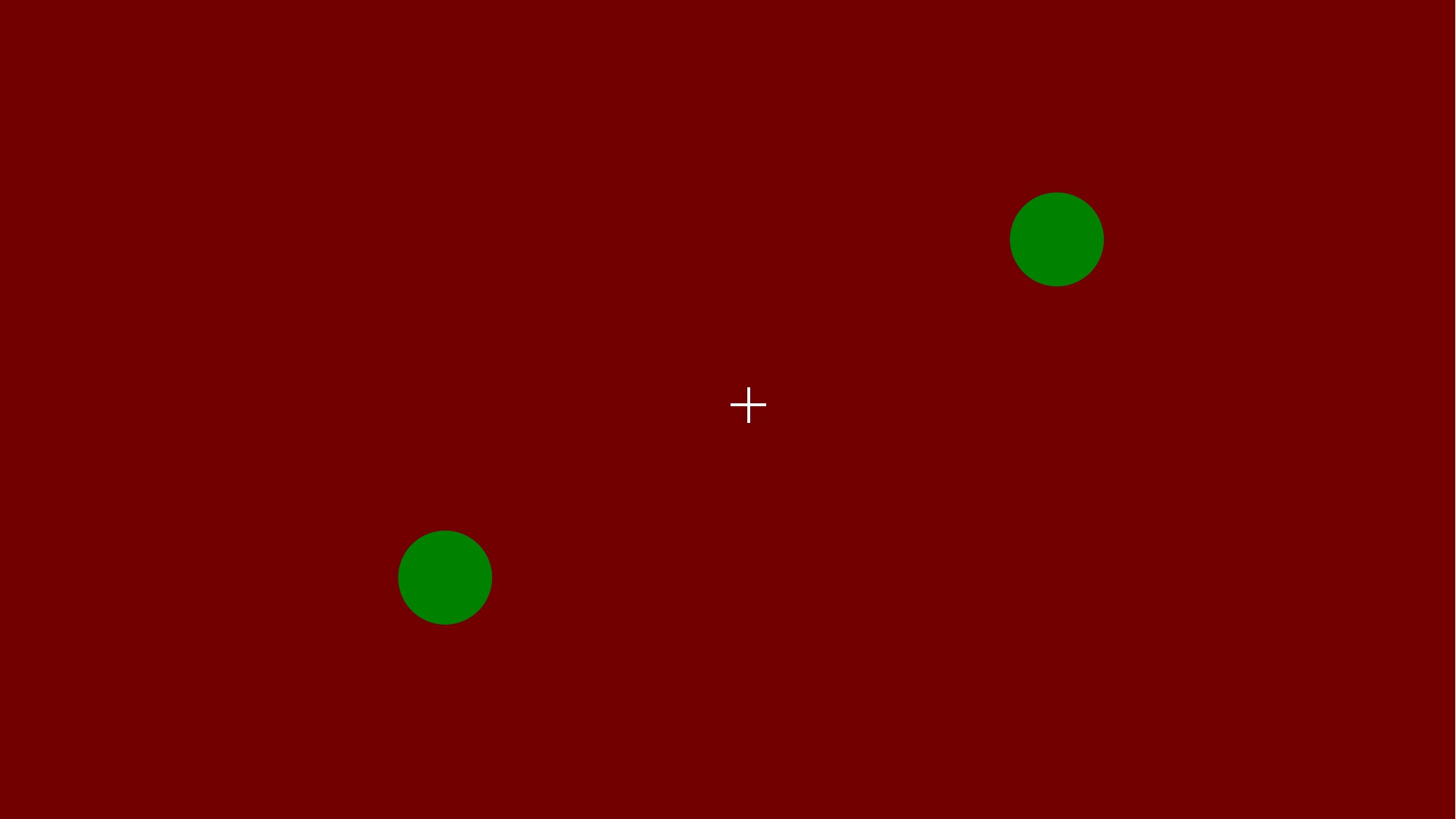

## Slide 30
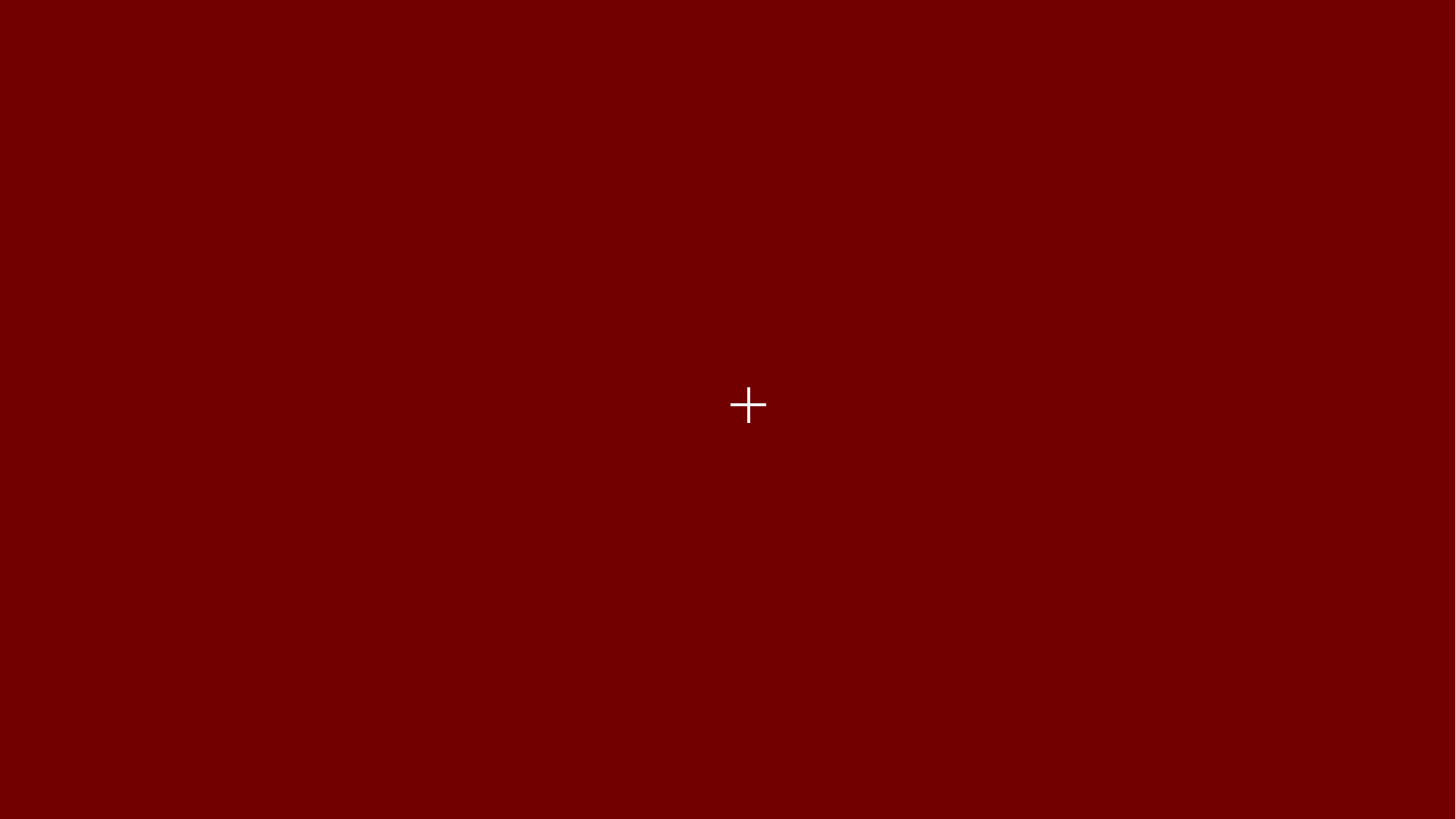

## Slide 31
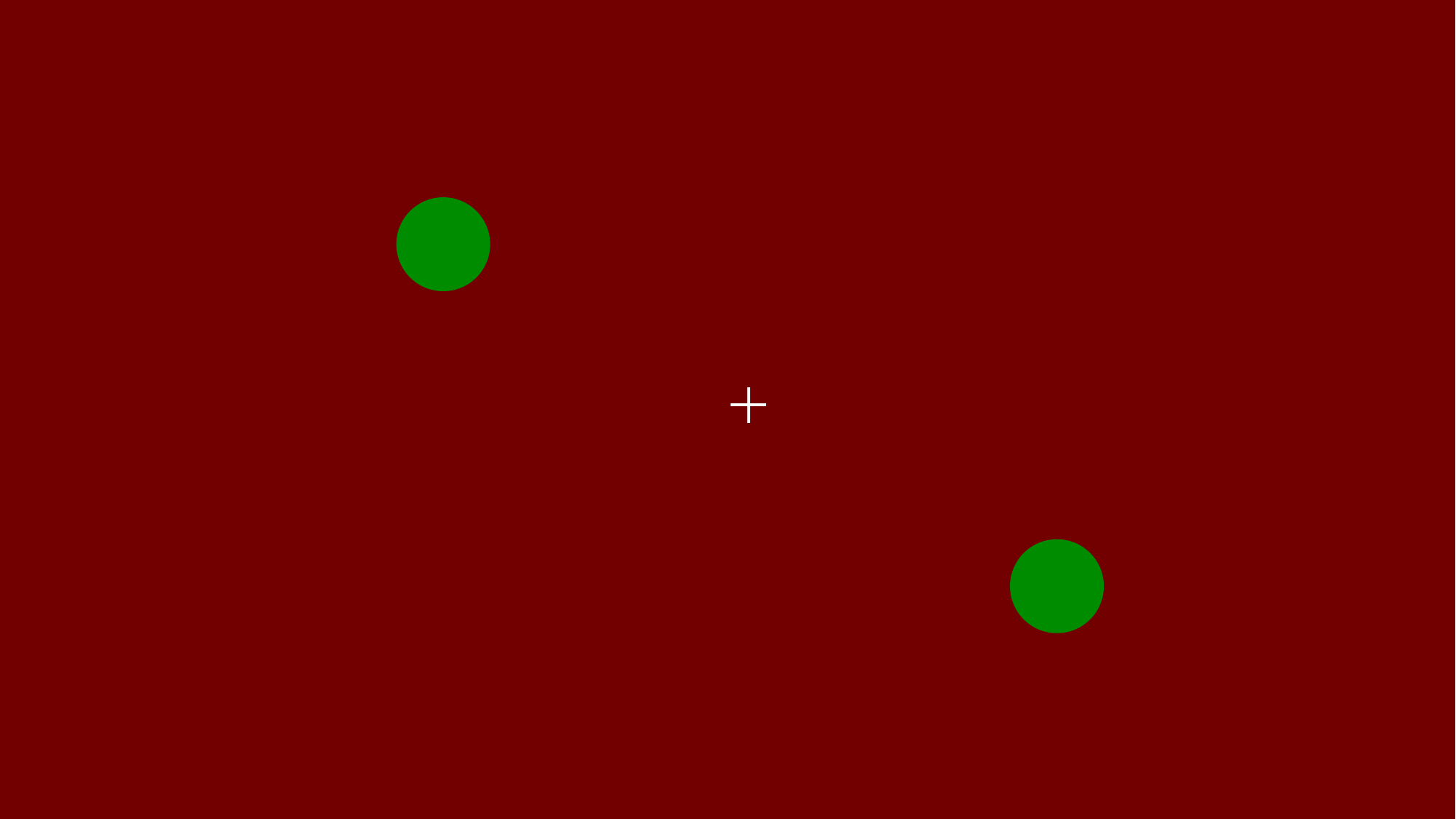

## Slide 32
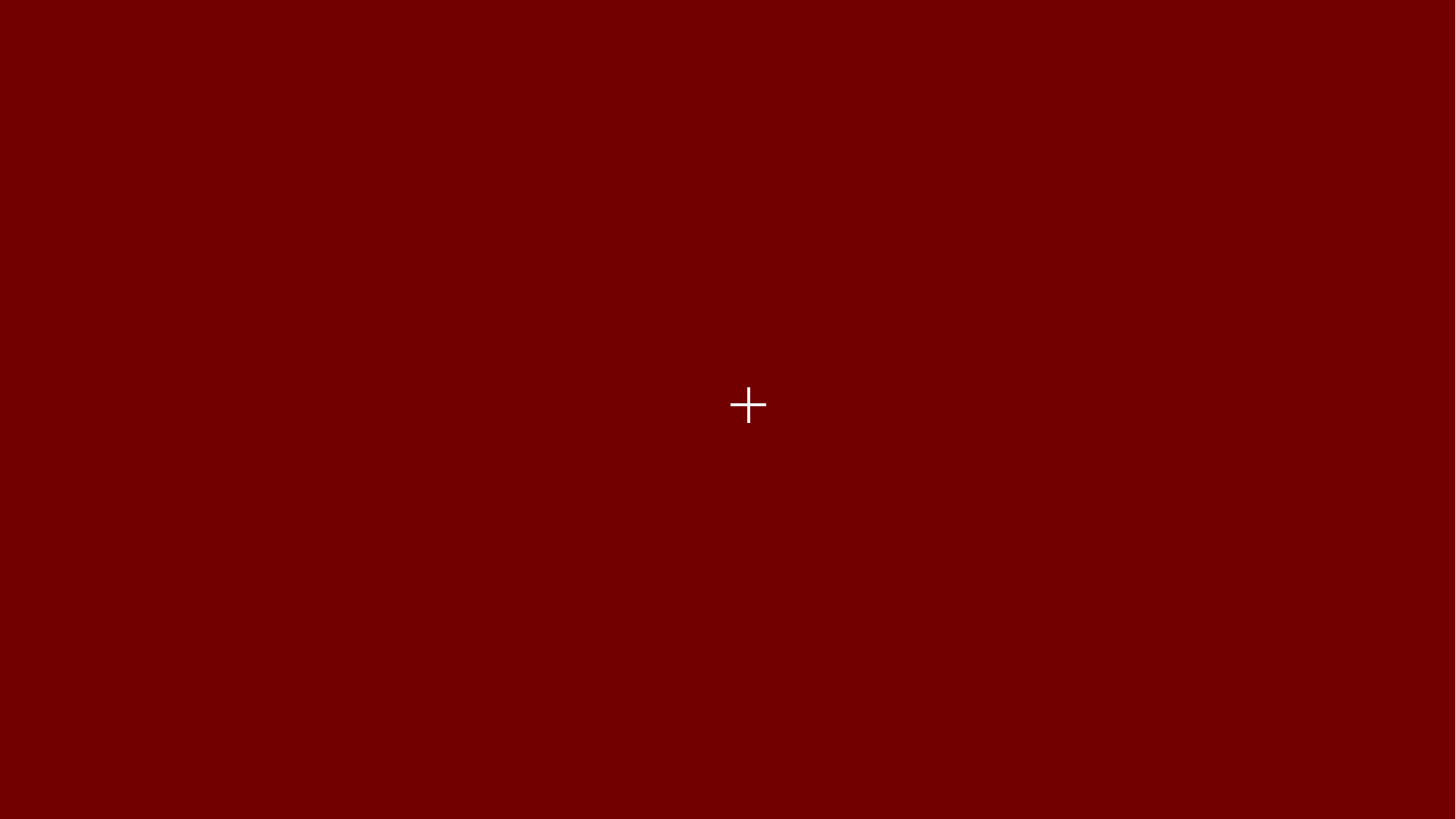

## Slide 33
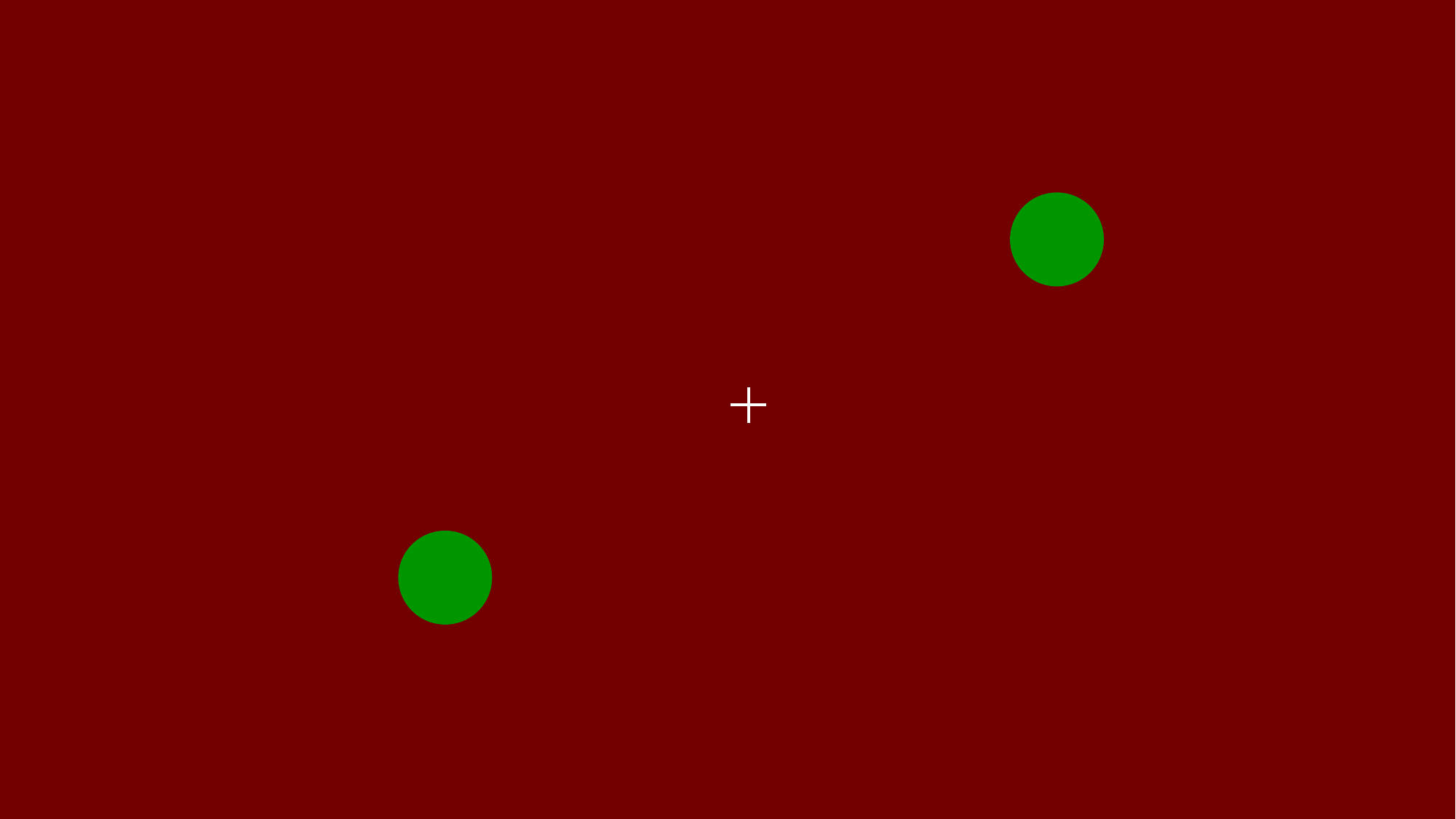

## Slide 34
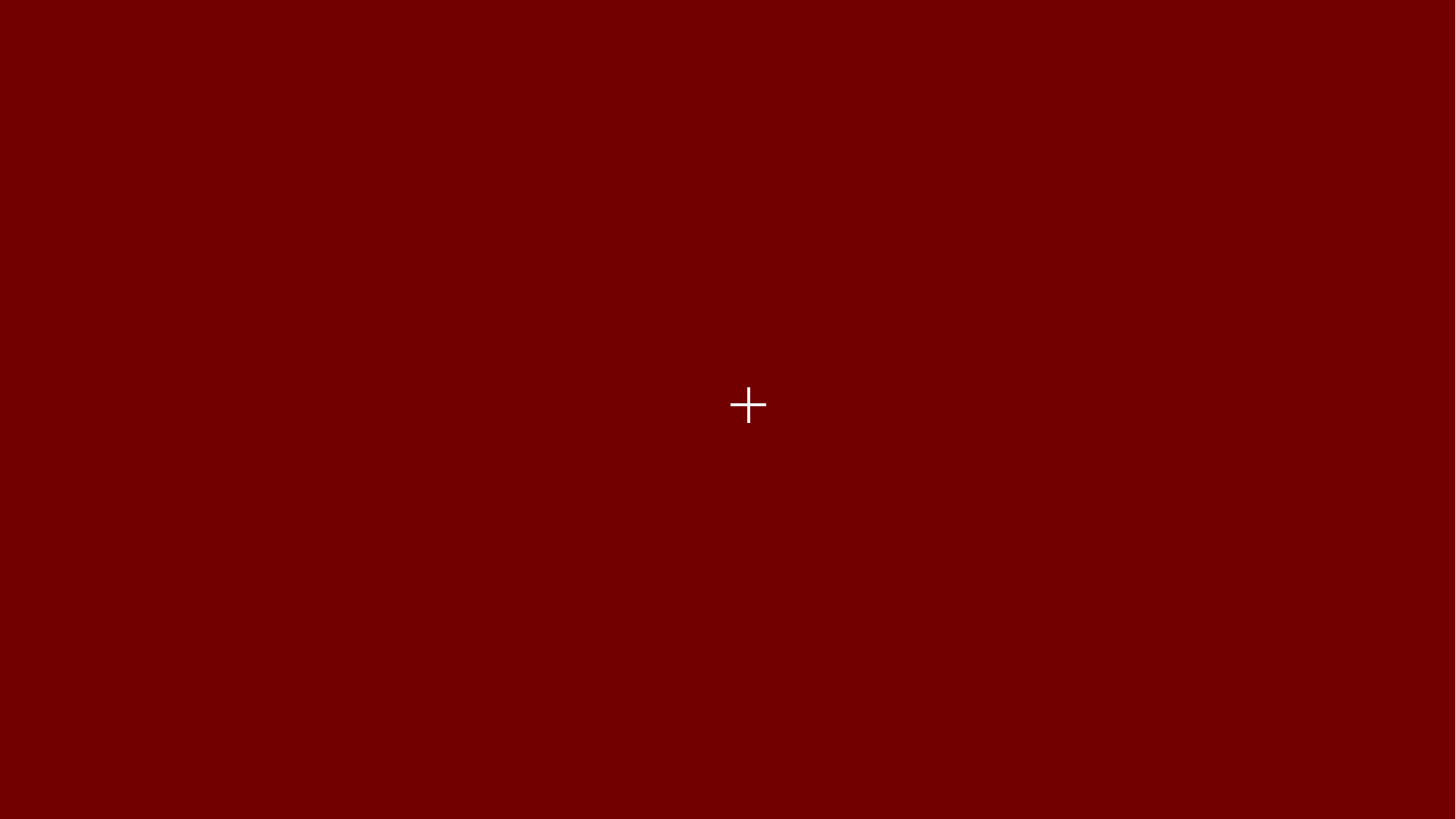

## Slide 35
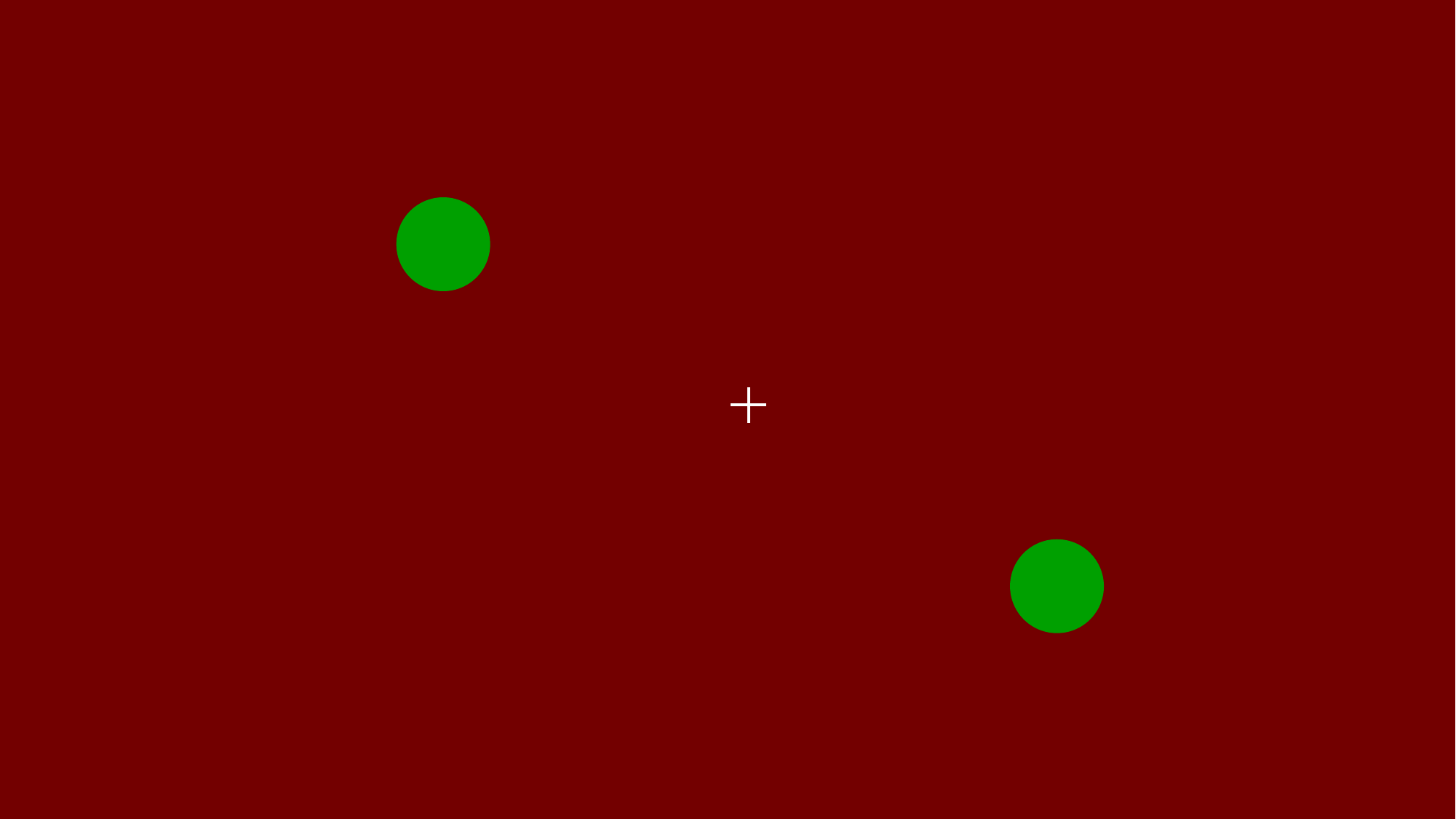

## Slide 36
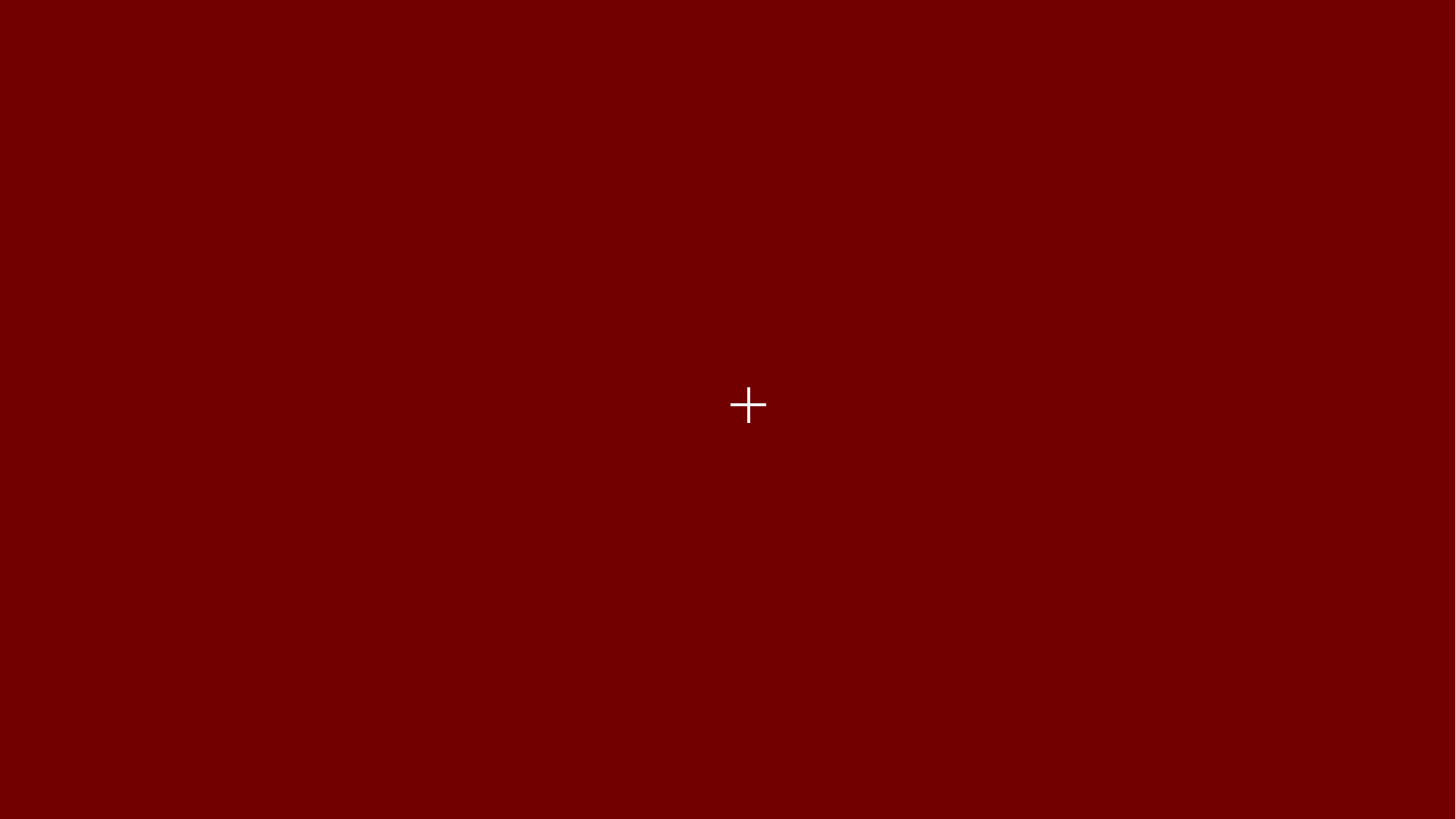

## Slide 37
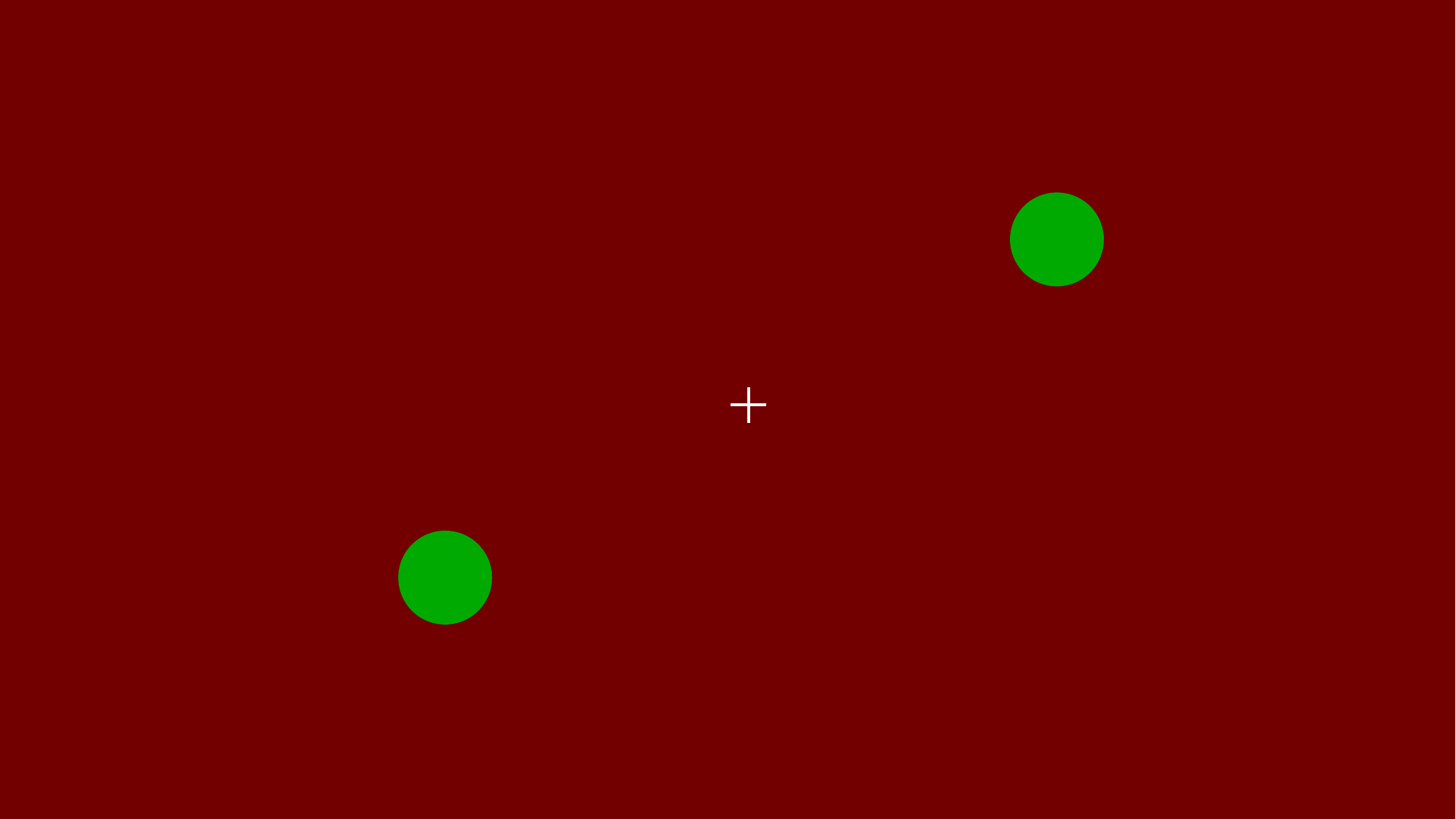

## Slide 38
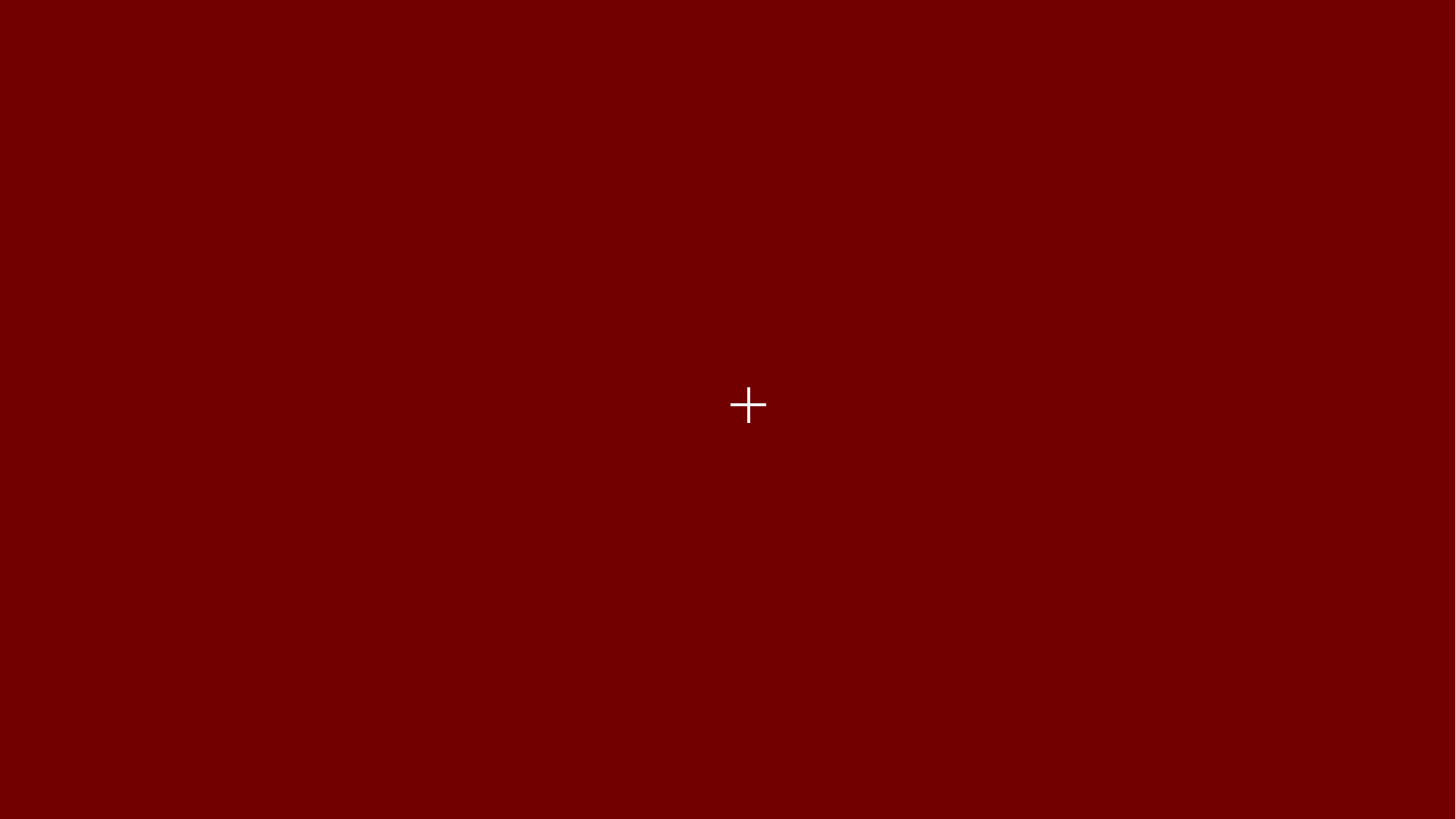

## Slide 39
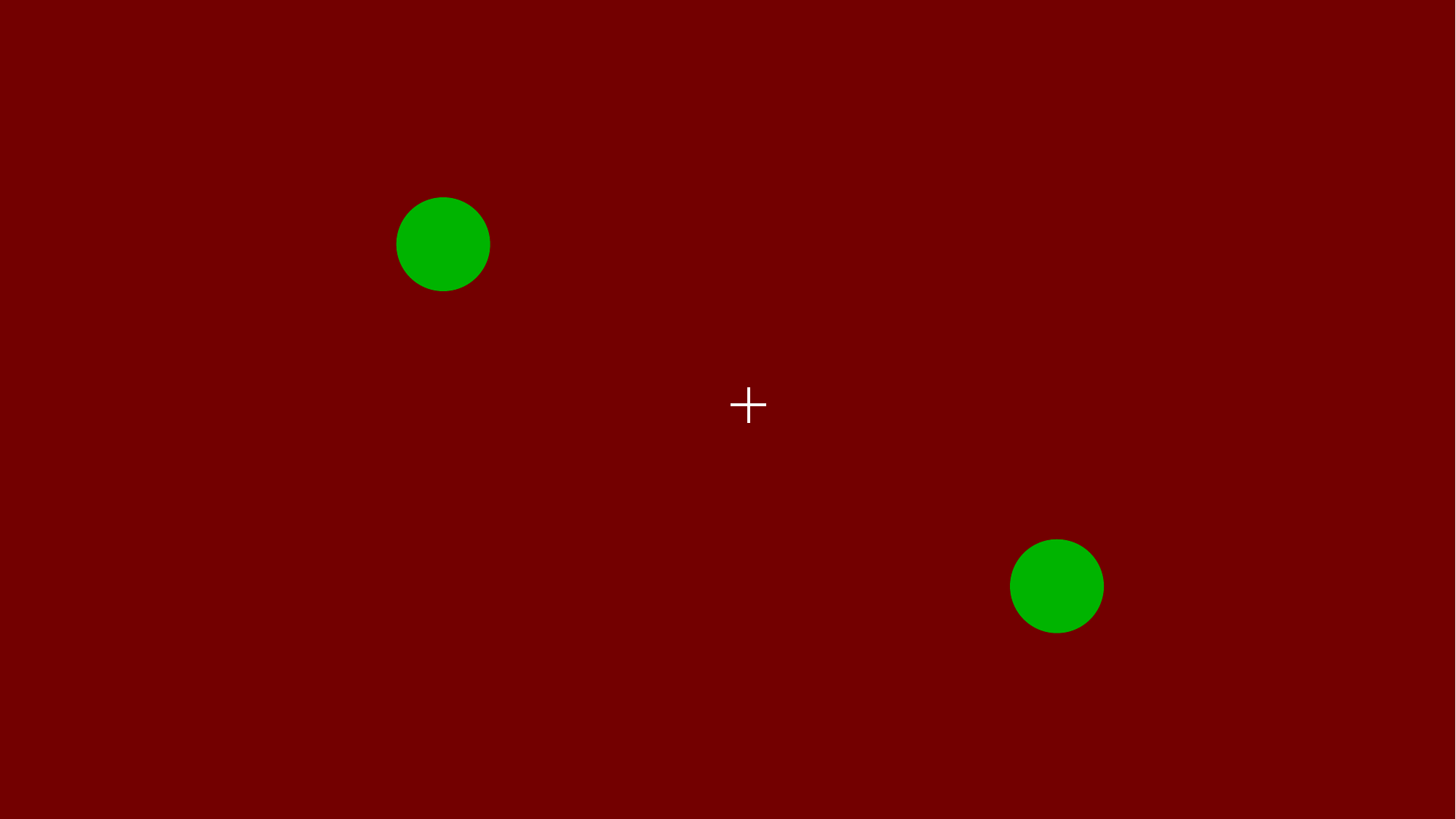

## Slide 40
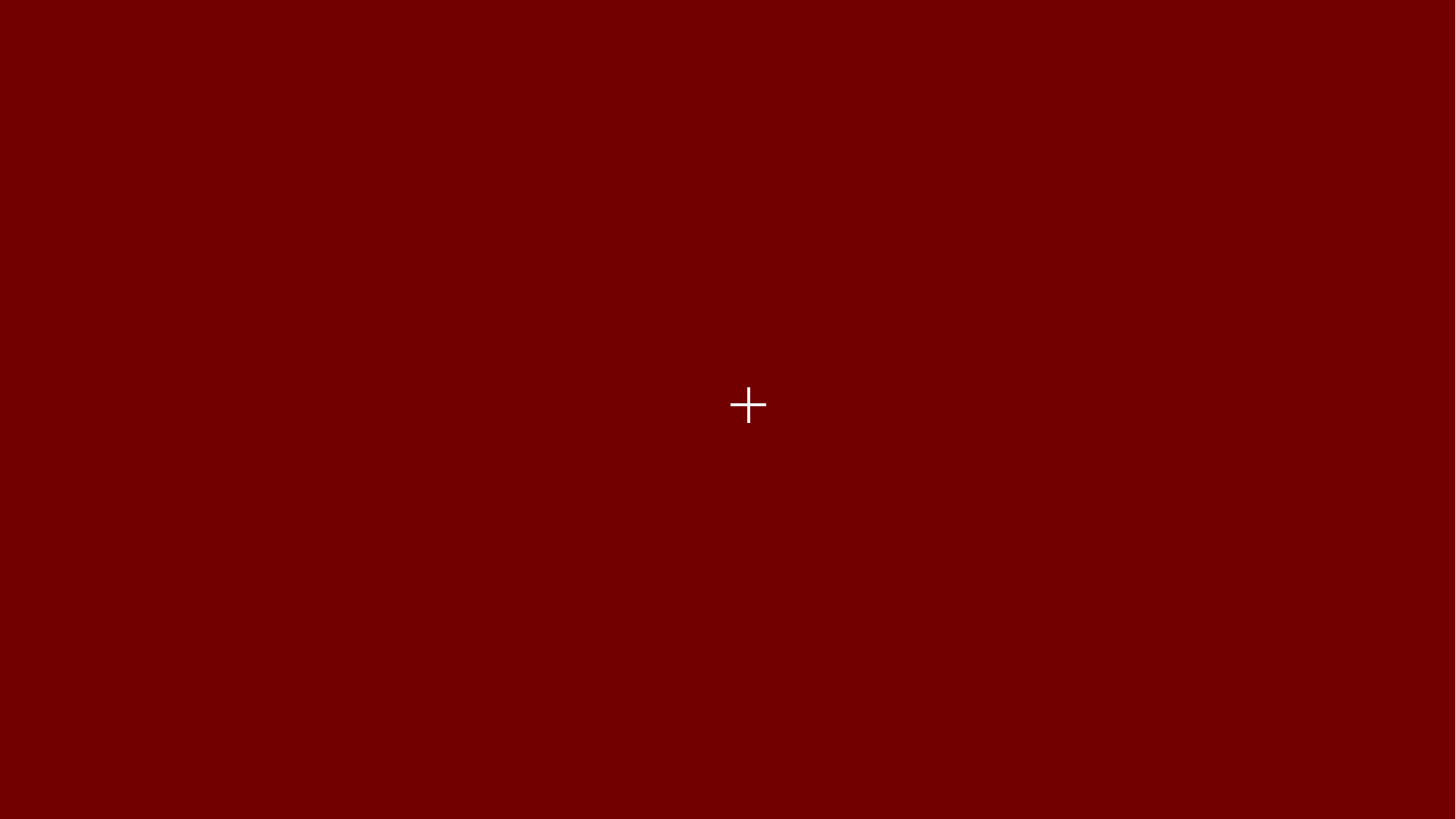

## Slide 41
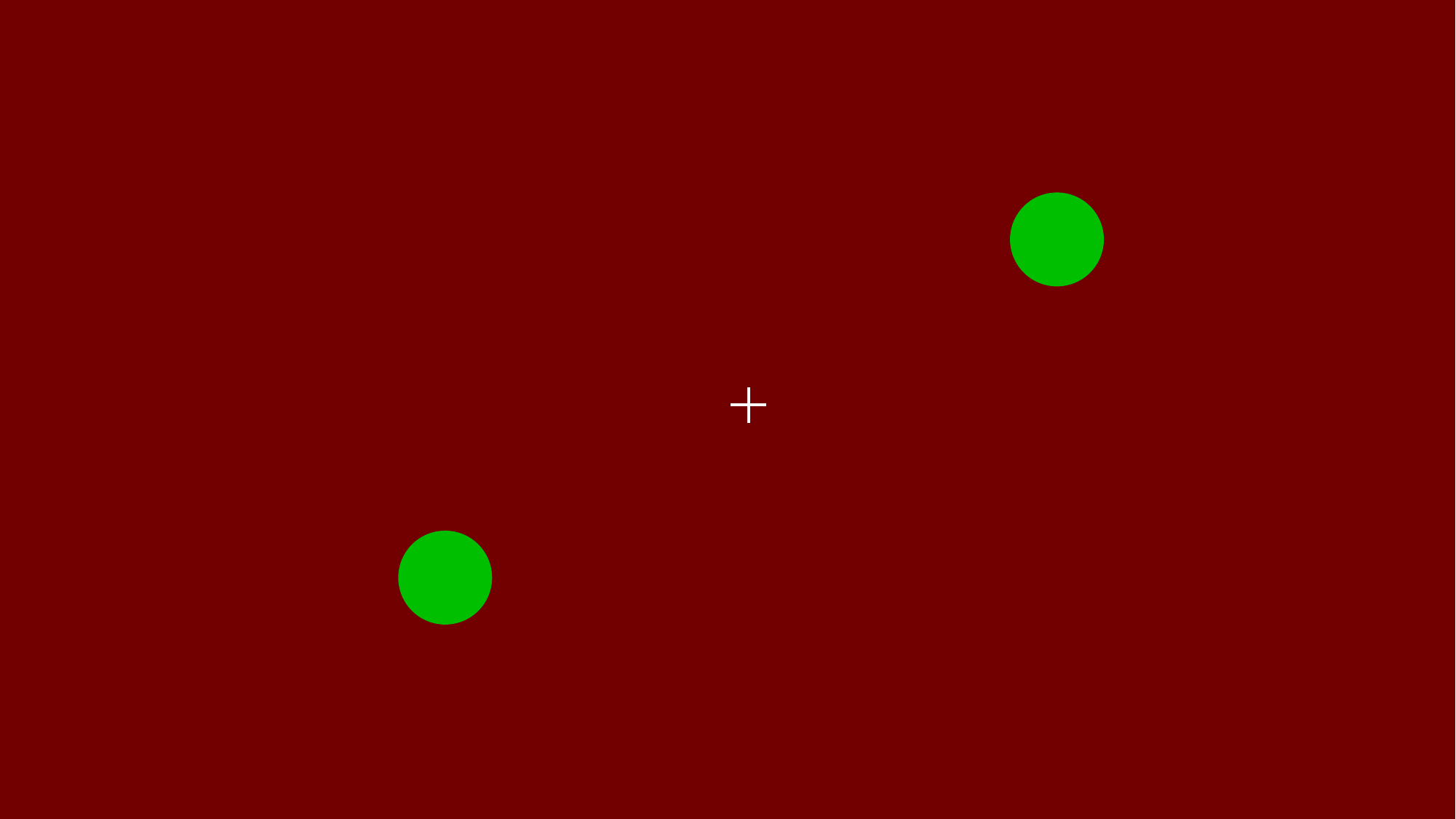

## Slide 42
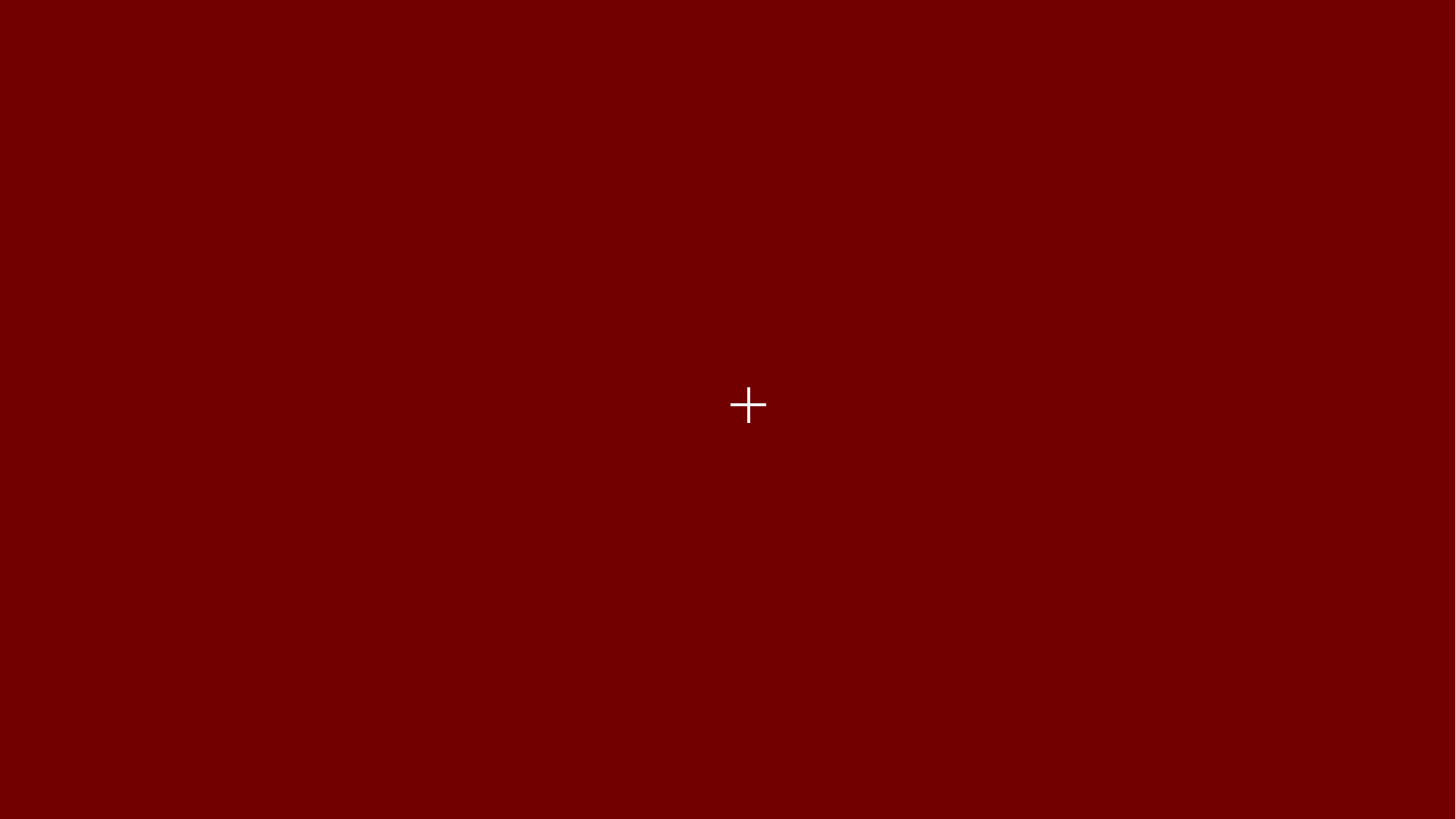

## Slide 43
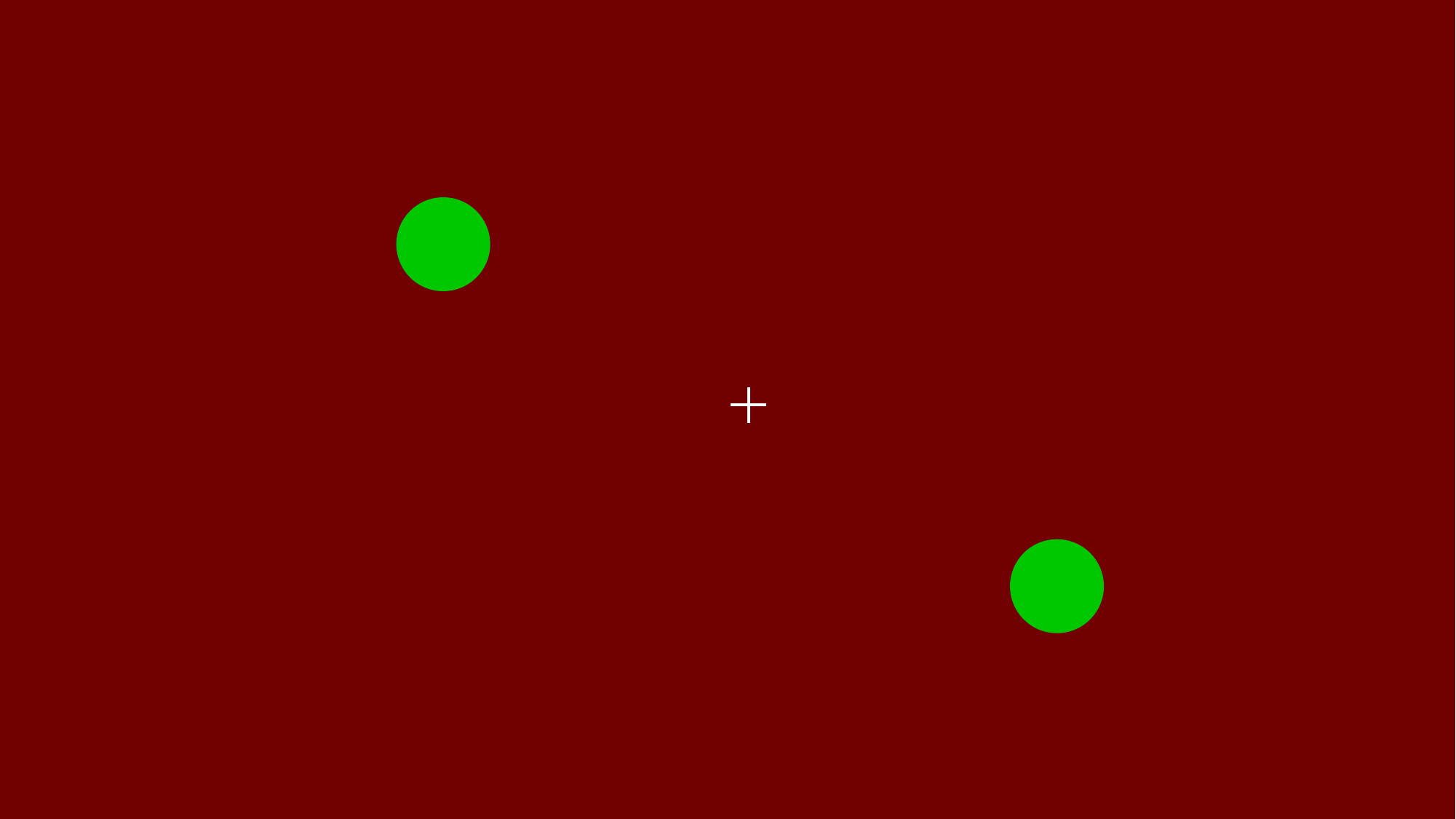

## Slide 44
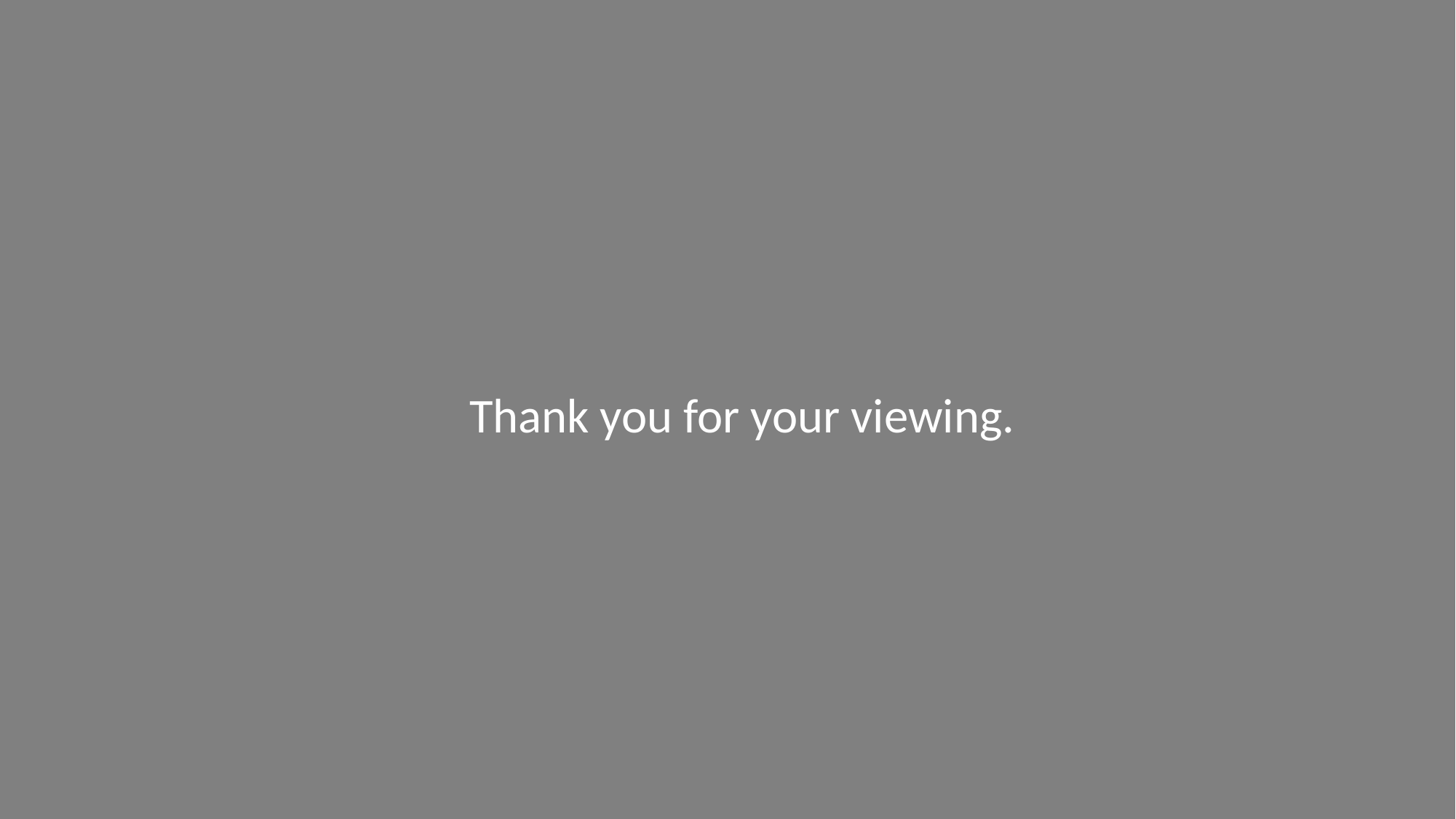

Thank you for your viewing.
